# Exploring the role of interoception in anxious traits and symptoms

**DOI:** 10.3389/fpsyt.2026.1769315

**Published:** 2026-04-29

**Authors:** Lucy Snell, Steven Reynolds, Matthew Garner, Gaby Pfeifer, Jayne Morriss

**Affiliations:** 1 School of Psychology, Faculty of Environmental and Life Sciences, University of Southampton, Southampton, United Kingdom; 2 School of Medicine and Population Health, University of Sheffield, Sheffield, United Kingdom

**Keywords:** anxiety, anxiety sensitivity, heartbeat perception, interoception, intolerance of uncertainty, trait anxiety

## Abstract

**Introduction:**

Interoception, the ability to sense internal bodily signals, has been increasingly linked to anxiety, yet the mechanisms underlying this relationship remain largely unknown. This study explored the associations between multiple dimensions of interoception and anxiety-related traits and symptoms in a non-clinical adult sample.

**Methods:**

A total of 305 participants completed self-report measures assessing interoceptive beliefs, attention, and perceived interoceptive accuracy, alongside measures of anxiety-related traits and symptoms. A subsample (n = 103) additionally completed laboratory-based heartbeat perception tasks to assess interoceptive accuracy, confidence, and insight via heartbeat counting and detection tasks..

**Results:**

Correlational analyses revealed that anxiety-related traits were significantly associated with self-reported interoceptive difficulties. Novel associations between intolerance of uncertainty and interoception were observed, including negative correlations with interoceptive insight, bodily trust, and increased tendency to worry about discomforting internal sensations. Anxiety symptom severity was linked to self-reported increased attention to bodily signals, reduced bodily trust, and lower perceived accuracy. However, no significant relationships were observed between the heartbeat perception task measures and anxiety-related traits, symptoms, or self-reported interoceptive abilities..

**Discussion:**

Findings support the conceptualisation of interoception as a multidimensional construct, demonstrating interoceptive accuracy and interoceptive beliefs as distinct constructs. Results align with emerging evidence that anxious traits are more closely related to subjective beliefs and interpretations of bodily signal than to behavioural interoceptive accuracy. These findings contribute to theoretical models of anxiety by highlighting interoceptive beliefs and attentional processes, rather than accuracy, as key factors warranting further investigation in clinical contexts.

## Introduction

Interoception refers to the nervous system’s ability to sense, interpret, and regulate signals from within the body (1, 2). It plays a critical role in maintaining homeostasis (3, 4) and has been found to underpin a range of higher-order cognitive functions, including attention, perception, decision-making, memory, and emotion regulation (5, 6). Given its role within these core cognitive and physiological processes, interoception has been increasingly studied as a transdiagnostic mechanism underlying the pathophysiology of various mental health conditions (7).

Suksasilp and Garfinkel (8) proposed a multidimensional framework of interoception that expands on previous dimensional models (9), by incorporating a broader range of dimensions and distinguishing them based on levels of processing. At the lowest level, the framework considers the fundamental strength and nature of afferent bodily signals, followed by their preconscious impact and neural representation. Higher-order conscious dimensions of interoception include interoceptive accuracy, beliefs, insight, attention, and the attribution of interoceptive sensations (8). Interoceptive accuracy is defined as the ability to correctly identify internal body signals, measured by behavioural tests of interoceptive accuracy such as heartbeat perception and detection tasks (10, 11). While cardiac tasks are widely used as a measure of interoceptive accuracy, interoception extends across other bodily systems, including respiratory and gastric domains, which have also been linked to anxiety in lab-based tasks of accuracy (12).

Interoceptive beliefs refers to one’s own perceptions and expectations regarding bodily sensations, measured through self-report measures such as questionnaires and confidence ratings. Interoceptive insight is a metacognitive measure which reflects how accurate individuals are at detecting their internal body signals, measured by the relationship between behavioural task performance (e.g., accuracy) and self-report performance (e.g. confidence). Interoceptive attention refers to the degree of focus to interoceptive sensations, typically assessed using self-report questionnaire measures, such as the Body Perception Questionnaire (BPQ; 13). Lastly, the attribution of interoceptive sensations refers to how individuals interpret bodily signals, such as perceiving them as threatening or ambiguous, which can influence emotional and behavioural responses (8).

Interoceptive processes have been increasingly linked to anxiety and may represent a transdiagnostic mechanism across anxiety-related disorders (14). Converging neurophysiological evidence supports this association: studies using heartbeat−evoked potentials (HEPs) show altered cortical processing of cardiac signals in individuals with generalized anxiety disorder (GAD), including reduced condition−dependent modulation and heightened HEP amplitudes related to symptom severity (15), as well as exaggerated HEP responses during adrenergic stimulation (16).

Complementing this neurophysiological work, research has begun to explore this through individual differences in anxious traits and symptom severity across anxiety disorders (17–19). Hierarchical models of anxious traits position trait anxiety as a central higher order dimension which is underpinned by several lower-order dimensions, such as intolerance of uncertainty and anxiety sensitivity (20).

The relationship between trait anxiety and facets of interoception is highly heterogeneous within the literature. Some studies report positive correlations between trait anxiety and cardiac interoceptive accuracy (21–23) whilst others have found a negative relationship (24, 25), or no relationship (26–29). Meta-analyses have similarly found no consistent association between cardiac interoceptive accuracy and trait anxiety (30, 31).

In contrast, the relationship between trait anxiety and interoceptive beliefs has been more consistently reported, with studies using the Multidimensional Assessment of Interoceptive Awareness (MAIA) as a measure of interoceptive beliefs showing negative associations with trait anxiety (28, 32–34). Specifically, Mehling (35) identified that subscales related to regulating attention, not-worrying about, and trusting bodily sensations consistently show the strongest negative associations though some studies report no significant relationships (36). Evidence regarding interoceptive attention is similarly mixed, with some studies identifying heightened bodily awareness as a predictor of trait anxiety (37) and others reporting no relationship (38). Additionally, Harrison et al. (39) found elevated trait anxiety was linked to decreased interoceptive insight in women, but not in men, indicating a potential gender-specific association. Together, these inconsistencies highlight the need for a multidimensional approach to examining interoception in relation to trait anxiety.

Anxiety sensitivity (AS) refers to the fear of anxiety-related bodily sensations and the belief that these sensations are harmful (40). The relationship between AS and interoception has yielded inconsistent findings, with studies reporting positive, negative, and null associations (21, 41–45), likely reflecting methodological variability in interoceptive measurement. Regarding interoceptive beliefs, AS has been negatively associated with the ‘Not Worrying’, ‘Attention Regulation’, and ‘Trusting’ subscales of the MAIA (34, 38), though findings remain mixed (46, 47). Given that AS is inherently centred on the interpretation of bodily sensations, further investigation into its relationship with multiple dimensions of interoception is warranted.

Intolerance of uncertainty (IU) reflects an aversive response to uncertainty and has been identified as a transdiagnostic factor underpinning anxiety (18). Despite recent advances in understanding interoception, the relationship between IU and interoception has not been well defined in literature (48). However, a recent correlation analysis by Bijsterbosch et al. (49) identified a weak positive association between IU and the ‘Emotional Awareness’ subscale on the MAIA, suggesting lower levels of IU were associated with higher levels of Emotional Awareness. Additionally, IU showed negative associations with ‘Not Distracting’, ‘Not Worrying’, and ‘Trusting’ subscales (49). Beyond these findings, empirical research directly linking IU and interoception is limited, although there is emerging research linking them theoretically; Freeston and Komes (50) provide a theoretical account of how IU may be conceptualised as a “felt sense” or embodied experience of unsafety. Given IU’s established role as a transdiagnostic factor in anxiety, empirical investigation into its relationship with multiple dimensions of interoception remains a notable gap in the literature.

Understanding how trait anxiety, anxiety sensitivity and IU interact with different aspects of interoception (as outlined in the Multidimensional Framework; 8) could clarify transdiagnostic mechanisms through which interoceptive processes contribute to symptom expression and maintenance. Emerging perspectives highlight the transdiagnostic role of interoception across anxiety disorders, including generalised anxiety disorder (GAD) (15), obsessive-compulsive disorder (OCD) (51), panic disorder (52), social anxiety disorder (SAD) (53), post-traumatic stress disorder (PTSD) (54, 55), as well as depression (56). However, these relationships remain unclear and understudied, with existing findings often yielding inconsistent results across anxiety-related disorders.

Given this transdiagnostic relevance, improving our understanding of the role of interoception may offer novel insights into the early identification and treatment of emotional disorders (2, 57). However, much of the existing research has focused on comparing clinical groups with control groups rather than adopting a dimensional approach that examines symptom severity across populations. This limits our understanding of how interoceptive processes contribute to anxiety-related psychopathology along a continuum. Addressing these gaps is particularly pertinent given the rising global rates of anxiety disorders (58).

The present study aimed to explore the relationship between interoceptive dimensions (accuracy, attention, beliefs and insight) and transdiagnostic anxious traits, including trait anxiety, anxiety sensitivity and intolerance of uncertainty, using both self-reported and task-based interoceptive measures. The study additionally examined these associations in relation to anxiety-related symptoms across GAD, OCD, panic disorder, PTSD, and SAD. This was guided by the following research questions:

1. How do anxiety-related traits relate to self-reported interoception?
  Based on prior research, trait anxiety and anxiety sensitivity were expected to be positively associated with interoceptive attention (BPQ). Both were also predicted to show negative associations with interoceptive beliefs, particularly the *Not Worrying*, *Not Distracting*, *Self-Regulation*, and *Trusting* subscales of the MAIA.
2. How do anxiety-related symptoms relate to self-reported interoception?
  Given mixed findings in the literature, associations between anxiety symptoms (GAD, OCD, panic, PTSD, social anxiety, depression) and self-reported interoceptive attention and beliefs were examined exploratorily, without directional predictions.
3. How do anxiety-related traits relate to task-based interoception?
  Due to inconsistent evidence regarding the relationship between anxiety-related traits (trait anxiety, anxiety sensitivity, intolerance of uncertainty) and interoceptive accuracy, confidence, or awareness, these analyses were considered exploratory.
4. How do anxiety-related symptoms relate to task-based interoception?
  Given limited and inconsistent findings regarding symptomatology and task-based interoceptive performance, these associations were also examined without directional predictions.

## Methods

The present study comprised of two independent groups: an online sample and a lab-based sample. The online group completed a series of questionnaires remotely, while the lab-based group completed the same questionnaires in-person and additionally completed two heartbeat perception tasks. Ethical approval was granted by the ethics committee of the University of Southampton (ERGO number 89229), and the study was conducted in accordance with the Declaration of Helsinki. All participants provided informed consent prior to participation and were advised that their participation was voluntary and that they could withdraw at any time without penalty. No deception was used in this study. All data were anonymised and stored securely in accordance with data protection guidelines.

### Participants

#### Recruitment

Participants were recruited using a combination of convenience and voluntary response sampling. Online participants were recruited via social media platforms (e.g., LinkedIn). Lab-based participants were recruited through social media platforms and the University of Southampton’s research participation system (SONA). SONA participants received course credit as compensation for their time and effort. Participants not recruited through SONA received no compensation.

#### Participant inclusion and exclusion criteria

Inclusion criteria required participants to be 18 years of age or older, and to have sufficient fluency in English to complete the questionnaires and tasks, which was self-assessed by the participants. Exclusion criteria included being under 18 years of age, lacking sufficient English fluency, completing less than 85% of the questionnaires and tasks, having a history of traumatic head injury, or currently taking psychotropic medication, as such medications may alter subjective and psychophysiological responses. Current or past mental health diagnoses were not used as explicit exclusion criteria, as the study was designed to reflect a general non-clinical population rather than a screened clinical sample. Participants recruited through the student research panel (SONA) confirmed their eligibility via an initial screening, while those recruited externally confirmed their eligibility through email before participating in the study.

#### Participant characteristics

Power analysis was conducted using G*Power V3.1.9.2 (59). A-priori power analysis estimated a minimum sample size of approximately *N* = 84 adult participants for completing heartbeat perception tasks and questionnaires in the lab. The sample size was calculated using a bivariate normal model for correlation analyses (effect size = 0.30, α error probability = 0.05, power (1 - β) = 0.80). For the online questionnaire group, a larger sample size of *N* = 193 was estimated using a smaller effect size (*r* = 0.20) to ensure sufficient power for detecting more subtle relationships. This larger sample size also accounts for potential incomplete questionnaire data. The effect sizes were based on prior studies examining intolerance of uncertainty processes and interoceptive measures (e.g., 60, 61).

**Figure 1:**
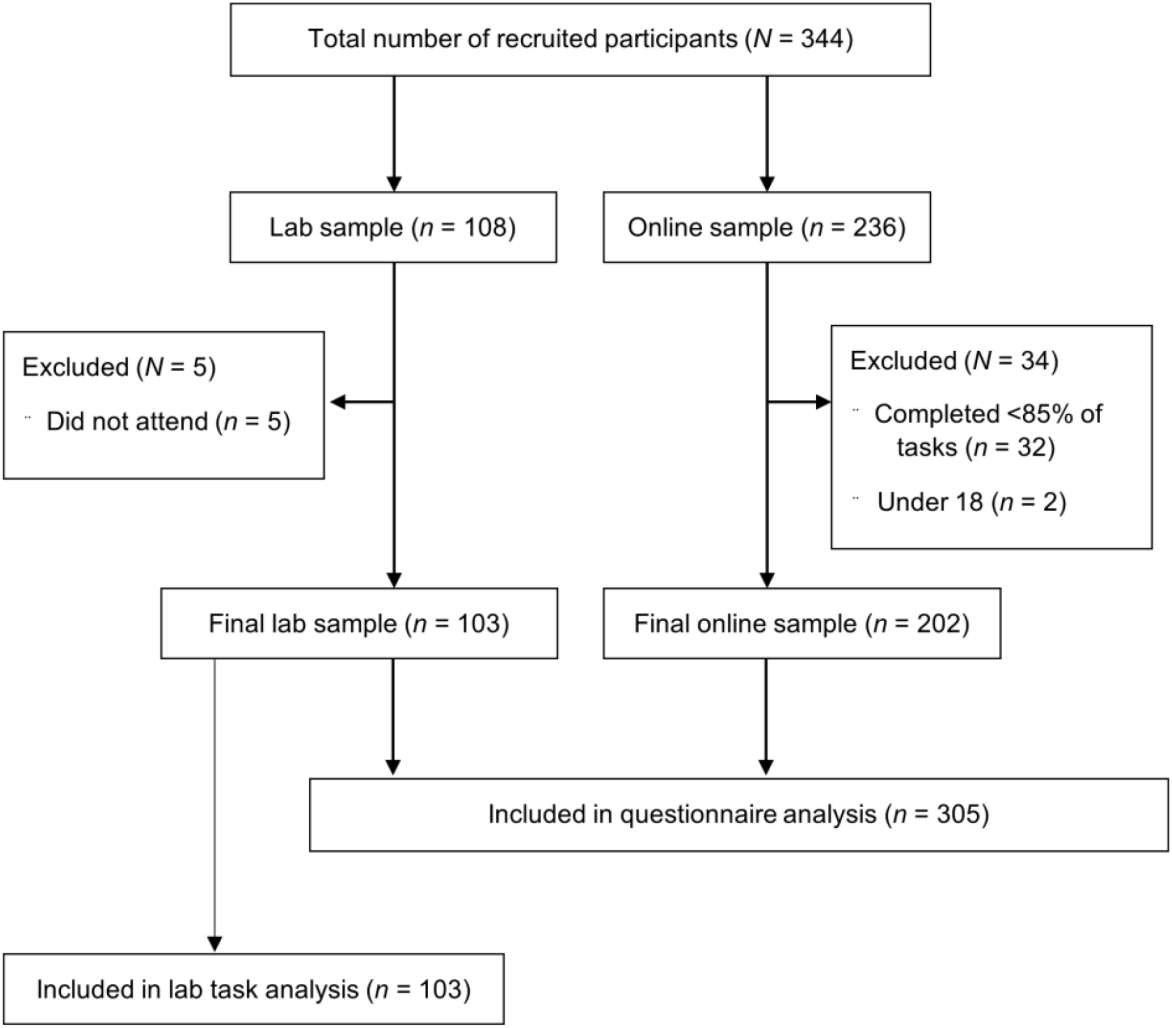
Participant flow diagram.

A total of 344 participants were recruited to take part across the online and lab-based components of the study. Of the 344 participants recruited, 39 participants did not meet eligibility criteria and were therefore excluded. The final sample consisted of 305 participants, including 202 online participants and 103 lab-based participants (see **Figure 1**). The mean age of the overall sample was 28 years old (*SD* = 13.32; ranging from 18 to 81) and 74.8% were female. For further demographic characteristics of each group, see **Table 1**.

**Table 1:** Participant demographic information by group.

	Lab group (*n* = 103)	HH Online group (*n* = 202)
Mean age (*SD*)	21.25 (5.28)	31.97 (14.87)
Gender		
Female	84 (81.6%)	144 (71.3%)
Male	19 (18.4%)	47 (23.3%)
Transgender	0 (0%)	1 (0.5%)
Prefer not to say	0 (0%)	10 (5%)
Ethnicity		
Asian	9 (8.7%)	11 (5.4%)
Black	5 (4.9%)	4 (2%)
Multiethnic	3 (2.9%)	4 (2%)
White	83 (80.6%)	155 (76.7%)
Other	3 (2.9%)	13 (6.5%)
Prefer not to say	0 (0%)	15 (7.4%)
Sexual Orientation		
Asexual	0 (0%)	2 (1%)
Bisexual	16 (15.5%)	21 (10.4%)
Heterosexual	78 (75.7%)	146 (72.3)
Homosexual	7 (6.8%)	9 (4.5%)
Prefer not to say	2 (1.9%)	24 11.9%)

#### Online participant characteristics

A total of 236 participants completed the online questionnaires using a data collection platform, Qualtrics. Thirty-two cases were excluded due to partial completion of the questionnaire, and a further two were excluded due to not reaching the minimum age requirement. Within the final online sample of 202 participants, 96% completed all questionnaires, while 4% of participants completed at least 87% of the 12 questionnaires (i.e., at least 9 questionnaires). The final online sample had a mean age of 31.97 years (SD = 14.87), with 71.3% identifying as female.

#### Lab participant characteristics

A total of 108 participants were recruited to complete questionnaires and heartbeat perception tasks in the laboratory. Five participants did not attend their scheduled timeslot, resulting in a final lab sample of 103 participants. The final lab sample had a mean age of 21.25 (*SD* = 5.28), with 81.6% identifying as female.

### Measures

Participants completed a series of validated self-report measures to assess anxiety-related traits, anxiety-related symptoms, and interoceptive beliefs and attention. Demographic data related to age, sex, ethnicity, nationality, English language fluency and sexual orientation was also collected. The questionnaires outlined below were administered with both online and lab-based participants. No modifications were made to the original scales.

#### Anxiety trait measures

Intolerance of Uncertainty Scale - 12 (IUS-12; 62**).** The IUS-12 is a 12 item self-report questionnaire intended to measure intolerance of uncertainty. Items include questions such as *“Unforeseen events upset me greatly”* and “*The smallest doubt can stop me from acting*” which are rated on a 5-point Likert scale ranging from 1 *(not at all characteristic of me)* to 5 (*entirely characteristic of me)*. Scores range from 12 to 60, with higher scores indicating greater intolerance of uncertainty. The scale has demonstrated excellent internal consistency in prior research (α = .85–.91; 62).

State-Trait Anxiety Inventory – Trait Subscale (STAIT-5; 63**).** The STAIT-5 is self-report questionnaire used to assess an individual’s general tendency to experience anxiety. It has five items derived from the trait-anxiety subscale of the original 20-item *Spielberger State-Trait Anxiety Inventory* – Trait Subscale (STAI-T) (64). Participants rate the frequency of anxiety-related feelings (e.g., *“I feel nervous and restless”*) on a 4-point Likert scale ranging from 1 (*Almost never*) to 4 (*Almost always*). Total scores range from 6 to 24, with higher scores indicating greater trait anxiety. The measure has demonstrated excellent internal consistency (α = .91; 63).

Anxiety Sensitivity Index (ASI; 40**).** The ASI is a 16-item self-report questionnaire used to assess the fear of anxiety-related bodily sensations, such as a racing heart or difficulty breathing. Items are rated on a 5-point Likert-type scale ranging from 0 (*very little)* to 4 (*very much*). Total scores range from 0 to 64, where higher scores indicate greater anxiety sensitivity. Sample items include *“When I feel tense, I worry that I might be seriously ill”* and *“It scares me when my heart beats rapidly”*. The ASI has demonstrated good internal consistency and construct validity (65).

#### Anxiety symptom measures

Patient Health Questionnaire (PHQ-9; 66). The PHQ-9 is a nine-item self-report measure for assessing depressive symptoms. Participants rate how often they have been bothered by symptoms such as *“Feeling down, depressed, or hopeless”* over the past two weeks on a 4-point Likert scale ranging from 0 (*Not at all*) to 3 (*Nearly every day*). Total scores range from 0 to 27, with higher scores indicating greater depressive symptom severity. A cut-off score of 10–14 is suggested for diagnosis of moderate depression. The PHQ-9 has demonstrated excellent internal consistency (α = .89; 66) and strong construct validity (67).

Generalised Anxiety Disorder Questionnaire (GAD-7; 68). The GAD-7 was used to measure symptoms of generalised anxiety. The GAD-7 is a widely used 7-item self-report questionnaire that assesses the frequency of anxiety symptoms over the past two weeks. Participants rate how often they have been bothered by difficulties such as *“Feeling nervous, anxious, or on edge”* on a 4-point Likert scale ranging from 0 (*Not at all*) to 3 (*Nearly every day*). Total scores range from 0 to 21, with higher scores indicating greater severity of anxiety. A cut-off score of 10–14 is suggested for diagnosis of moderate anxiety. The GAD-7 has demonstrated excellent internal consistency (Cronbach’s α = .92) and strong construct validity (68).

Obsessive Compulsive Inventory – Revised (OCI-R; 69**).** The OCI-R is an 18-item self-report questionnaire designed to assess distress associated with obsessive-compulsive symptoms. Participants rate how much they have been bothered or distressed by each symptom (e.g., *“I repeatedly check doors, windows, drawers, etc.”*) over the past month on a 5-point Likert scale ranging from 0 *(Not at all)* to 4 *(Extremely)*. Total scores range from 0 to 72, with higher scores indicating greater OCD symptom severity. A cut-off score of 21 is suggested for diagnosis-level symptomology. The OCI-R has demonstrated excellent internal consistency (Cronbach’s α = .90) and strong test-retest reliability (*r* = 0.84; 69).

Panic Disorder Severity Scale – Self Report (PDSS-SR; 70**).** The PDDS-SR is a 7-item self-report measure intended to measure the severity of panic disorder. It assesses various dimensions of panic disorder, including the frequency of panic attacks, anticipatory anxiety, agoraphobic avoidance, and functional impairment. Participants rate the severity of each symptom over the past week using a 5-point Likert scale ranging from 0 *(None)* to 4 *(Extreme*), for example *“If you had any panic attacks during the past week, how distressing were they while they were happening?”*. Total scores range from 0 to 28, with higher scores indicating greater severity of panic symptoms. A cut-off score of 8 is suggested for diagnosis-level symptomology. The PDSS has shown good internal consistency (α = 0.92 (70).

Post-traumatic Stress Disorder Checklist for DSM-5 (PCL-5; 71**).** The PCL-5 is a 20 item self-report questionnaire intended to measure the frequency of PTSD symptoms in the past month. Participants rate statements such as *“In the past month, how much were you bothered by having Repeated, disturbing, and unwanted memories of the stressful experience*?” using a 5-point Likert scale ranging grom 0 (*Not at all*) to 4 (*Extremely)*. The total score ranges from 0 to 80, with higher scores indicating more severe PTSD symptoms. A cut-off score of 31 is suggested for diagnosis-level symptomology. The PCL-5 has demonstrated high internal consistency (α = .94), and test-retest reliability (*r* = .82) (72).

Social Interaction Phobia Scale (SIPS; 73**).** The SIPS is a 14-item self-report questionnaire intended to measure symptoms of social anxiety symptoms. Participants rate how much they agree with statements such as *“I am nervous mixing with people I don’t know well”* on a 5-point Likert scale ranging from 0 (*Not at all*) to 4 (*Extremely*). Total scores range from 0 to 56, with higher scores indicating greater severity of social anxiety symptoms. There is no standard clinical cut-off for the SIPS measure, however, a score of 24 or above is considered clinically significant. The SIPS has demonstrated good internal consistency (73).

#### Self-report measures of interoception

Multidimensional Assessment of Interoceptive Awareness – Version 2 (MAIA-v2; 74**).** The MAIA-2 is a 37-item self-report scale intended to assess multiple facets of self-reported interoceptive beliefs. Participants rate statements such as “*I can pay attention to my breath without being distracted by things happening around me”* on a 6-point Likert scale ranging from 0 *(Never*) to 5 (*Always)*, with nine reverse-scored items. The MAIA-2 consists of eight subscales (outlined in **Table 2**), with mean scores calculated separately for each. Subscale scores range from 0 – 5. Higher scores indicate greater interoceptive awareness. The eight-factor structure has been validated across cultures in clinical (75), and non-clinical samples (76–78).

**Table 2:** MAIA-2 subscales (74).

MAIA subscale	Definition
Noticing	Awareness of bodily sensations
Not Distracting	Tendency not to ignore or distract from sensations of discomfort
Not Worrying	Tendency not to worry about discomforting sensations
Attention Regulation	Ability to sustain and control attention to bodily sensations
Emotional Awareness	Awareness of the connection between bodily sensations and emotions
Self-Regulation	Ability to regulate distress by attending to bodily sensations
Body Listening	Active listening to bodily signals for insight
Trusting	Experience of one’s body as safe and trustworthy

MAIA, Multidimensional Assessment of Interoceptive Awareness.

The MAIA-2 has demonstrated good internal consistency, with Cronbach’s α ranging from.64 (Noticing) to.83 (Attention Regulation and Trusting) (74). Test-retest reliability studies have shown moderate to good stability, with intraclass correlation coefficients ranging from.67 to.79 (78).

Body Awareness Subscale of the Body Perception Questionnaire-Short Form (BPQ-SF; 79). The BPQ-SF is a shorter version of the original full scale BPQ developed by Porges (13). The shorter form focuses primarily on the Body Awareness and Autonomic Reactivity subscales. In line with previous research, only the Body Awareness subscale was used in the present study given the focus on subjective awareness of bodily sensations (9, 80). This subscale is a 26 item self-report measure intended to assess an individual’s sensitivity to internal bodily sensations (79), relating specifically to the measure of interoceptive attention (8). Participants respond to statements such as *“During most situations, I am aware of how fast I am breathing”* on a 5-point Likert scale, ranging from 1 *(Never)* to 5 (*Always)*. Scores range from 26 – 130, with higher scores representing higher levels of body awareness. The psychometric properties of the BPQ Body Awareness scale demonstrated high internal consistency (α = .92) and high test-retest reliability (79).

Interoceptive Accuracy Scale (IAS; 81). The IAS is a 21-item self-report questionnaire designed to assess an individual’s perceived ability to accurately detect internal bodily signals. Participants rate statements such as *“I can accurately perceive when my heart rate changes”* on a 5-point Likert scale, ranging from 1 *(Strongly Disagree)* to 5 *(Strongly Agree*). Scores range from 21 to 105, with higher scores indicating greater self-reported interoceptive accuracy. Internal consistency has been found to be good, with Cronbach’s α ranging from.84 to.91 across studies (81).

#### Behavioural measures of interoception

**Figure 2:**
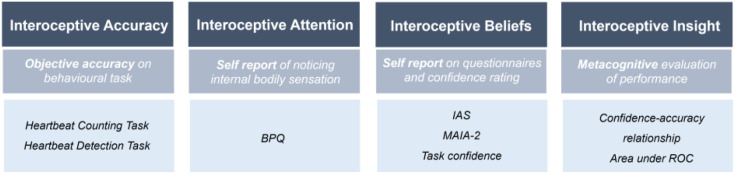
Overview of interoception dimensions and methods of measurement. Interoceptive terminology and methods of measurement, as outlined in Suksasilp & Garfinkel’s (2022) Multidimensional Model of Interoception. BPQ, Body Perception Questionnaire; IAS, Interoceptive Accuracy Scale; MAIA, Multidimensional Assessment of Interoceptive Awareness; ROC, Receiver Operating Characteristics.

Self-report questionnaires assessing interoception measure an individual’s perceived sensitivity to internal bodily signals; however, they do not determine whether this interoceptive belief corresponds to lab-based tasks of interoceptive accuracy (8, 9). Therefore, a smaller sample of participants (*n* = 103) completed heartbeat perception tasks to assess interceptive accuracy. These tasks were combined with a measure of subjective confidence in performing the task to produce an index of interoceptive insight. The relationship between mean task accuracy (interoceptive accuracy) and mean confidence scores (interoceptive beliefs) was analysed to quantify interoceptive insight scores. An overview of the interoceptive dimensions explored and methods of measurement is outlined in **Figure 2**.

##### Heartbeat counting task

In the Heartbeat Counting Task (HCT), participants were instructed to estimate their own heartbeats without external verification. At the start of each trial, an auditory cue *(“Start”)* was played through headphones, prompting participants to silently count their heartbeats without physically checking their pulse. At the end of the trial, a second cue *(“Stop”)* signalled them to stop counting and report their estimated heartbeat count. There were six trials, with intervals of 25, 30, 35, 40, 45, and 50 seconds, presented in a randomised order.

At the end of each trial, participants rated their confidence of their perceived accuracy. This confidence judgement was made by marking on a continuous visual analogue scale (VAS) that was 10 centimetres long. One end was marked “*Total guess/No heartbeat awareness*” while the other end was labelled “*Complete confidence/Full perception of heartbeat”*). Confidence ratings on the VAS were measured manually in millimetres using a ruler for each of the six trials and then averaged to produce a single participant confidence score of their heartbeat counting performance accuracy. Verbatim task instructions are provided in the **Supplementary Material**.

**Figure 3:**
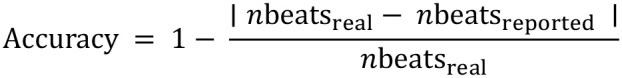
Interoceptive accuracy formula.

To measure interoceptive accuracy, a probability accuracy score, 0 to 1, was calculated for each trial, where higher scores indicated greater heartbeat counting accuracy. To provide a symmetric accuracy response between actual number of beats (*n*beats_real_) and reported number of beats (*n*beats_reported_), the absolute difference between these values was calculated using the formula outlined in **Figure 3**.

**Figure 4:**
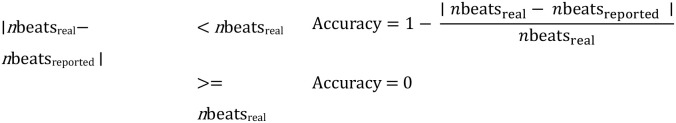
Amended interoceptive accuracy formula.

When the difference between *n*beats_real_ and *n*beats_reported_ is larger than *n*beats_real_, equation one can produce negative accuracy scores. Not only do these have no interpretable meaning but will cause erroneous mean accuracy values for the whole trial. To prevent this, the absolute difference in values was restricted to a maximum of *n*beats_real_, as given by the formula outlined in **Figure 4**. After six trials were completed the mean accuracy value was determined for each participant. Interoceptive insight was indexed by calculating within-participant correlations (*r*) between each participant’s accuracy score and their corresponding VAS confidence score (9).

##### Heartbeat detection task

In the Heartbeat Detection Task (HDT), participants were presented with auditory tones through headphones that were either synchronous or asynchronous with their own heartbeat. In the synchronous condition, auditory tones were delayed by 300 milliseconds to account for the average 250 milliseconds delay between the R-wave and the arrival of the pulse pressure wave at the finger (82). In the asynchronous condition, tones were delayed by an additional 300 milliseconds (i.e., approximately 550 milliseconds after the R-wave), making them perceptually out of sync with the heartbeat (83). The task comprised 20 trials, each lasting 20 seconds. This number of trials was selected to balance statistical power and participant fatigue (84, 85). After each trial, participants were asked to decide whether the auditory tones were synchronous or asynchronous with their own heart. They rated their confidence of their judgement on a 10-centimetre VAS ranging from 0 cm *(“Total guess/No heartbeat awareness”)* to 10 cm *(“Complete confidence/Full perception of heartbeat”).*

Confidence ratings on the VAS were measured manually in millimetres for each of the 20 trials and then averaged to produce a single confidence score. Interoceptive accuracy was determined by dividing the number of correct trials divided by the total number of trials, yielding the proportion of correct responses. As data from the HDT was binary, interoceptive insight (metacognitive awareness) was assessed using receiver operating characteristic (ROC) curve analysis (86). The area under the ROC curve quantified the extent to which confidence ratings reflected accuracy across trials (9).

### Procedure

#### Online group procedure

Questionnaire data was collected remotely using the survey platform Qualtrics (87). Participants accessed the survey via an online link distributed through the study advertisement. The advertisement included a brief study description, eligibility criteria, and a direct link to the survey. Within Qualtrics, participants were presented with the Participant Information Sheet, provided informed consent, and completed demographic questions before proceeding to the study measures. Upon completion, participants were provided with a debrief form within Qualtrics outlining the aims of the study and relevant contact and support information. The estimated completion time for all questionnaires was approximately 20 minutes.

#### Lab group procedure

**Figure 5:**
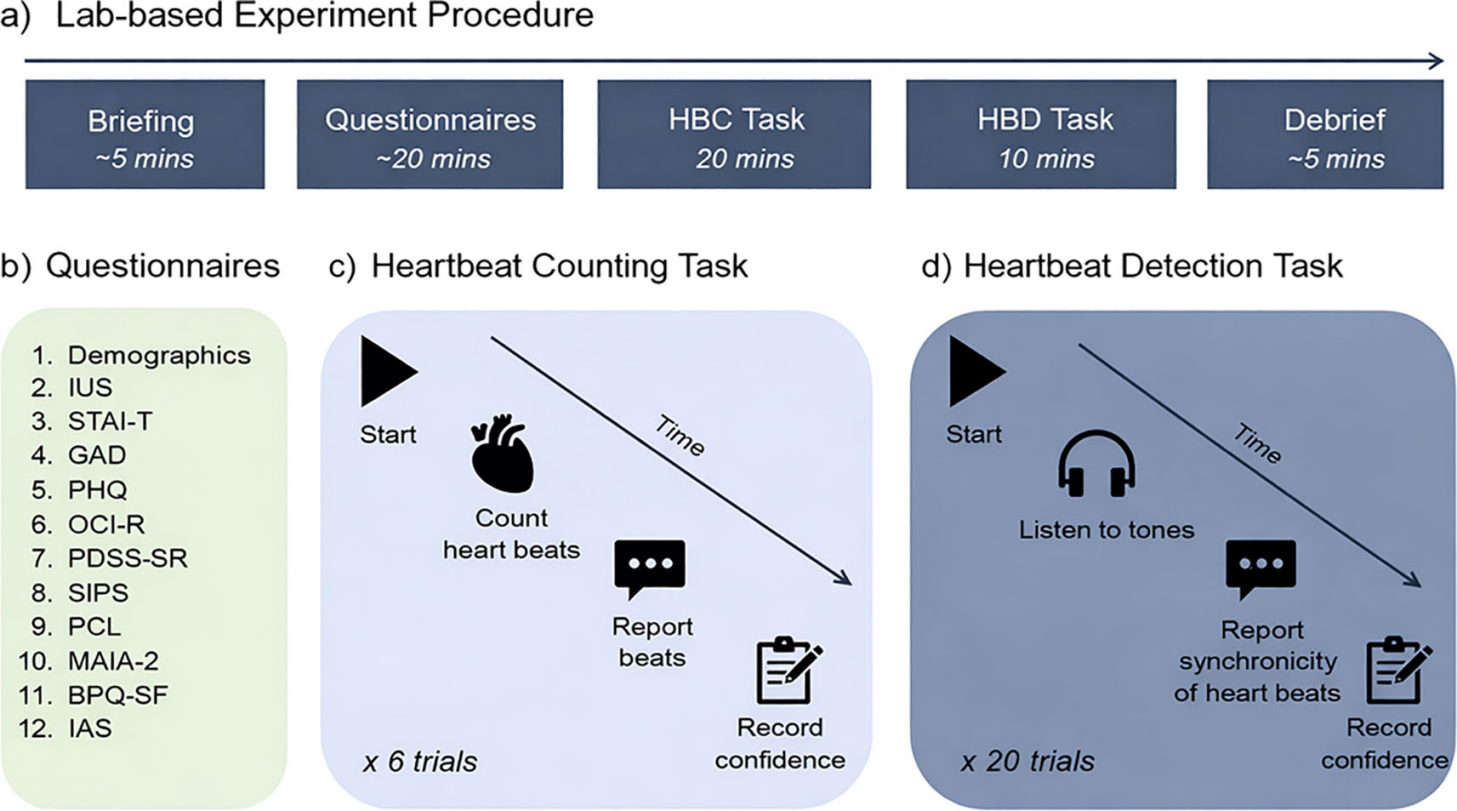
Laboratory-based experiment procedure **(a)**, Questionnaires **(b)**, Heartbeat Counting task **(c)**, and Heartbeat Detection Task. ASI, Anxiety Sensitivity Index; BPQ, Body Perception Questionnaire; GAD, Generalised Anxiety Disorder; HBC, Heartbeat Counting Task; HBD, Heartbeat Detection Task; IAS, Interoceptive Accuracy Scale; IUS, Intolerance of Uncertainty Scale; MAIA, Multidimensional Assessment of Interoceptive Awareness; OCI, Obsessive-Compulsive Inventory; PCL, PTSD Checklist; PDSS-SR, Panic Disorder Severity Scale – Short Version; PHQ, Patient Health Questionnaire; SIPS, Social Interaction Phobia Scale; STAI-T, State-Trait Anxiety Inventory – Trait.

Upon arrival at the laboratory, participants were provided with information regarding the experimental procedure. Participants were asked to review and sign an informed consent form to confirm their agreement to participate in the study. Participants were taken to the testing booth where they were asked to complete a series of questionnaires presented on a computer. Next, participants completed the heartbeat counting and heartbeat detection tasks. For these tasks, they were asked to wear headphones, and a reusable soft-sensor pulse oximeter (Nonin Medical XPOD^®^ 3012 LP with USB Connector) was placed on the index finger of their non-dominant hand to measure heartbeats. Tactile sensations from the pulse oximeter were minimised by using a soft, low-pressure sensor designed to minimise pulse-wave feedback (88). The pulse oximeter was connected to a PC and tracked heartbeats in real time during the counting task, allowing for an accurate comparison between actual and perceived counts. The heartbeat counting task was administered first to minimise potential confounds arising from prior exposure to one’s own heartbeat during the heartbeat detection task (as illustrated in **Figure 5**). The lab-based study took approximately 50 minutes to complete. Upon completion, participants were debriefed and provided with a written debrief form outlining the full aims of the study and relevant contact and support information.

### Data analysis

All experimental data were inputted and analysed using IBM SPSS version 29.0.2.0 (89). Prior to analysis the data set was examined for missing values, outliers and normality. The data were generally in line with normality assumptions based on skewness and kurtosis values, as well as from visualising histograms. Descriptive statistics and reliability analysis was carried out. Parametric correlational analyses were conducted to examine associations between anxiety trait measures, anxiety symptom measures, self-report interoceptive measures and heartbeat perception task performance. To control for multiple comparisons, false discovery rate (FDR) corrections were applied to correlation analyses using the Benjamini–Hochberg procedure with a false discovery rate set at *q* = .05.

Online and lab questionnaire data were combined (*N* = 305) to explore how subjective interoceptive measures correlate with transdiagnostic anxiety traits (i.e., trait anxiety, AS and IU) and clinical symptom questionnaires (i.e., GAD, OCD, panic disorder, PTSD, and SAD). In a smaller sub-group of participants who completed the heartbeat perception tasks (*n* = 103), task performance was correlated with the corresponding participant’s questionnaires to explore whether behavioural measures of interoception are associated with self-reported anxiety traits and symptoms.

### Exclusions and missing data

Data were screened for missing responses prior to analysis. All lab participants completed 100% of the questionnaires and heartbeat perception tasks. However, one participant’s data were missing for six trials due to a data recording issue in the Heartbeat Detection Task; their score was therefore averaged across the completed 14 trials rather than the full 20.

In the online sample, 236 participants initiated the questionnaire battery. Thirty-two participants (13.6%) were excluded for not meeting the pre-specified 87% completion threshold (i.e., at least 9 out of the 12 questionnaires). A further two participants were excluded for reporting an age below the eligibility criterion of 18 years. This resulted in a final online sample of 202 participants. Among these, all individual questionnaires had 100% item-level completion. However, eight online participants had partial missing data across the full battery: two completed 87%, four completed 92%, and two completed 96% of the total questionnaires. These participants were included in analyses for the measures they completed and excluded from analyses involving the measures they did not complete.

## Results

### Self-report measure descriptive statistics

**Table 3** presents descriptive statistics for the self-report measures in the online sample (*n* = 202). These measures demonstrated robust psychometric properties, with Cronbach’s α values ranging from.75 (*MAIA Not Worrying*) to.96 *(PCL)* for the online group. Most data approximated a normal distribution, although some measures exhibited positive skew. For instance, the PDSS-SR (skewness = 1.66) indicated a floor effect, consistent with the expectation that most participants in a non-clinical sample report low panic symptoms. Similarly, the OCI (skewness = 0.88) reflects lower levels of obsessive-compulsive symptoms within this sample.

**Table 3:** Descriptive statistics of online group questionnaires and subscales.

Questionnaires/Subscales	*N*	*M*	*SD*	Observed range	Skewness	Kurtosis	α
Trait Measures							
ASI	194*	26.81	13.06	0 – 59	0.28	-0.52	.91
IUS	202	33.20	9.56	13 – 57	0.18	-0.56	.91
STAIT-5	202	12.70	3.98	5 – 20	0.06	-0.99	.86
Interoceptive Measures							
BPQ	202	71.62	23.46	26 – 128	0.20	-0.52	.95
IAS	196*	44.85	13.10	21 – 92	0.46	0.42	.92
MAIA Noticing	202	2.57	1.28	0 – 5	-0.10	-0.67	.84
MAIA Not Distracting	202	2.25	1.13	0 – 5	0.21	-0.57	.87
MAIA Not Worrying	202	2.52	1.00	0 – 4.8	-0.08	-0.19	.75
MAIA Attention Regulation	202	2.36	1.14	0 – 5	0.05	-0.46	.91
MAIA Emotional Awareness	202	3.02	1.31	0 – 5	-0.49	-0.53	.90
MAIA Self-Regulation	202	2.22	1.27	0 – 5	0.19	-0.59	.89
MAIA Body Listening	202	1.91	1.27	0 – 5	0.33	-0.57	.88
MAIA Trusting	202	2.56	1.37	0 – 5	-0.15	-0.80	.88
Symptom Measures							
GAD-7	202	8.46	5.26	0 – 20	0.31	-0.75	.88
OCI	202	18.08	13.62	0 – 56	0.88	0.11	.92
PCL	202	23.14	18.73	0 – 75	0.69	-0.47	.96
PDSS-SR	202	3.96	5.23	0 – 28	1.66	3.01	.94
PHQ-9	202	8.57	6.33	0 – 26	0.62	-0.37	.89
SIPS	202	17.53	13.57	0 – 54	0.63	-0.46	.95

*N is reduced for ASI and IAS measures due to incomplete questionnaire responses. ASI, Anxiety Sensitivity Index; BPQ, Body Perception Questionnaire; GAD, Generalized Anxiety Disorder scale; IAS, Interoceptive Accuracy Scale; IUS, Intolerance of Uncertainty Scale; MAIA, Multidimensional Assessment of Interoceptive Awareness; OCI, Obsessive-Compulsive Inventory; PCL, PTSD Checklist; PDSS, Panic Disorder Severity Scale; PHQ, Patient Health Questionnaire; SIPS, Social Interaction Phobia Scale; STAI-T, State-Trait Anxiety Inventory – Trait. Cronbach’s α values represent internal consistency reliability for each group separately.

**Table 4** presents descriptive statistics for the self-report measures in the lab-based sample (*n* = 103). Internal consistency was similarly robust, with Cronbach’s α ranging from.74 *(MAIA Not Worrying)* to.96 (*SIPS*). Distributions for most measures were approximately normal; PDSS and OCI scores were positively skewed (skewness 1.01 and.93 respectively), similar to the online group.

**Table 4:** Descriptive statistics of lab group questionnaires and subscales.

Questionnaires/subscales	*N*	*M*	*SD*	Observed range	Skewness	Kurtosis	α
Trait Measures							
ASI	103	29.76	11.73	5 – 62	0.14	0.16	.88
IUS	103	32.29	9.13	14 – 58	0.38	-0.16	.91
STAIT-5	103	12.36	3.49	5 – 20	-0.03	-0.56	.84
Interoceptive Measures							
BPQ	103	72.58	21.16	35 – 130	0.44	-0.13	.94
IAS	103	78.76	11.61	35 – 105	-0.70	1.77	.89
MAIA Noticing	103	2.42	1.15	0 – 5	0.20	-0.73	.83
MAIA Not Distracting	103	2.17	0.94	0 – 4.67	0.08	-0.44	.87
MAIA Not Worrying	103	2.49	0.91	0.40 – 4.80	-0.08	-0.17	.74
MAIA Attention Regulation	103	2.37	0.90	0.43 – 4.43	0.03	-0.50	.85
MAIA Emotional Awareness	103	2.89	1.09	0.40 – 5	-0.17	-0.54	.84
MAIA Self-Regulation	103	2.26	0.95	0 – 4.25	-0.16	-0.18	.78
MAIA Body Listening	103	1.95	1.08	0 – 4.33	-0.04	-0.57	.83
MAIA Trusting	103	2.72	1.29	0 – 5	-0.18	-0.88	.90
Symptom Measures							
GAD-7	103	9.22	5.59	0 – 21	0.26	-0.95	.90
OCI	103	20.63	13.94	0 – 60	0.93	0.54	.92
PCL	103	26.90	17.07	0 – 60	0.05	-1.16	.93
PDSS-SR	103	4.88	4.99	0 – 23	1.02	0.52	.91
PHQ-9	103	9.76	6.48	0 – 26	0.41	-0.73	.88
SIPS	103	21.52	14.84	0 – 56	0.63	-0.34	.96

ASI, Anxiety Sensitivity Index; BPQ, Body Perception Questionnaire; GAD, Generalized Anxiety Disorder scale; IAS, Interoceptive Accuracy Scale; IUS, Intolerance of Uncertainty Scale; MAIA, Multidimensional Assessment of Interoceptive Awareness; OCI, Obsessive-Compulsive Inventory; PCL, PTSD Checklist; PDSS, Panic Disorder Severity Scale; PHQ, Patient Health Questionnaire; SIPS, Social Interaction Phobia Scale; STAI-T, State-Trait Anxiety Inventory – Trait. Cronbach’s α values represent internal consistency reliability for each group separately.

**Figure 6:**
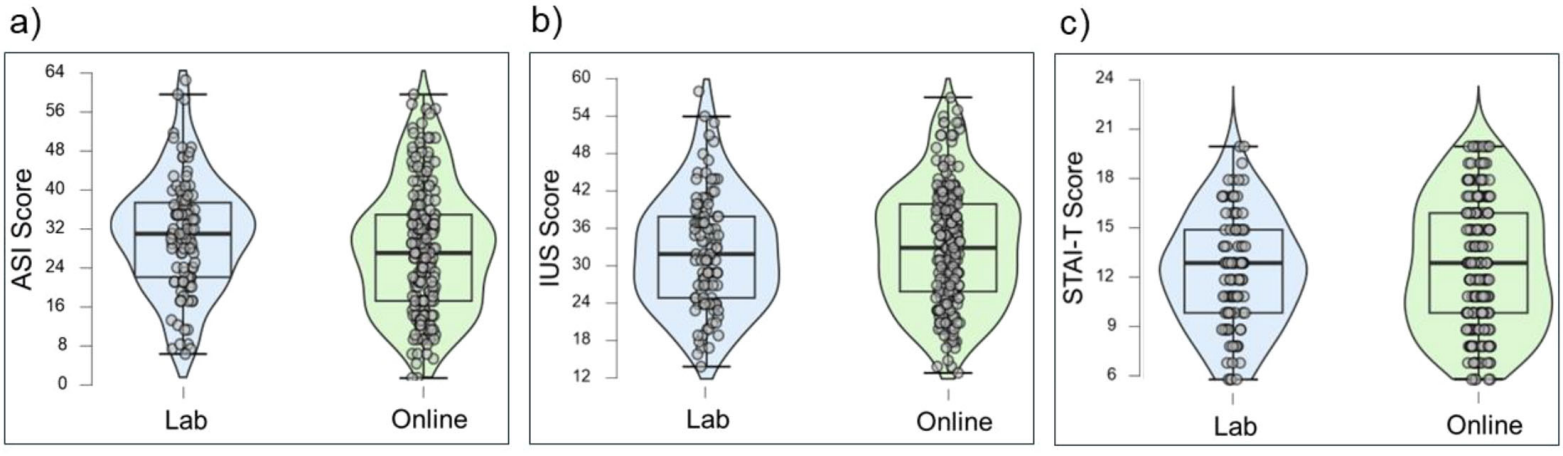
Violin plots of trait measures by group: ASI **(a)**, IUS **(b)** and STAI-T **(c)**.

**Figure 7:**
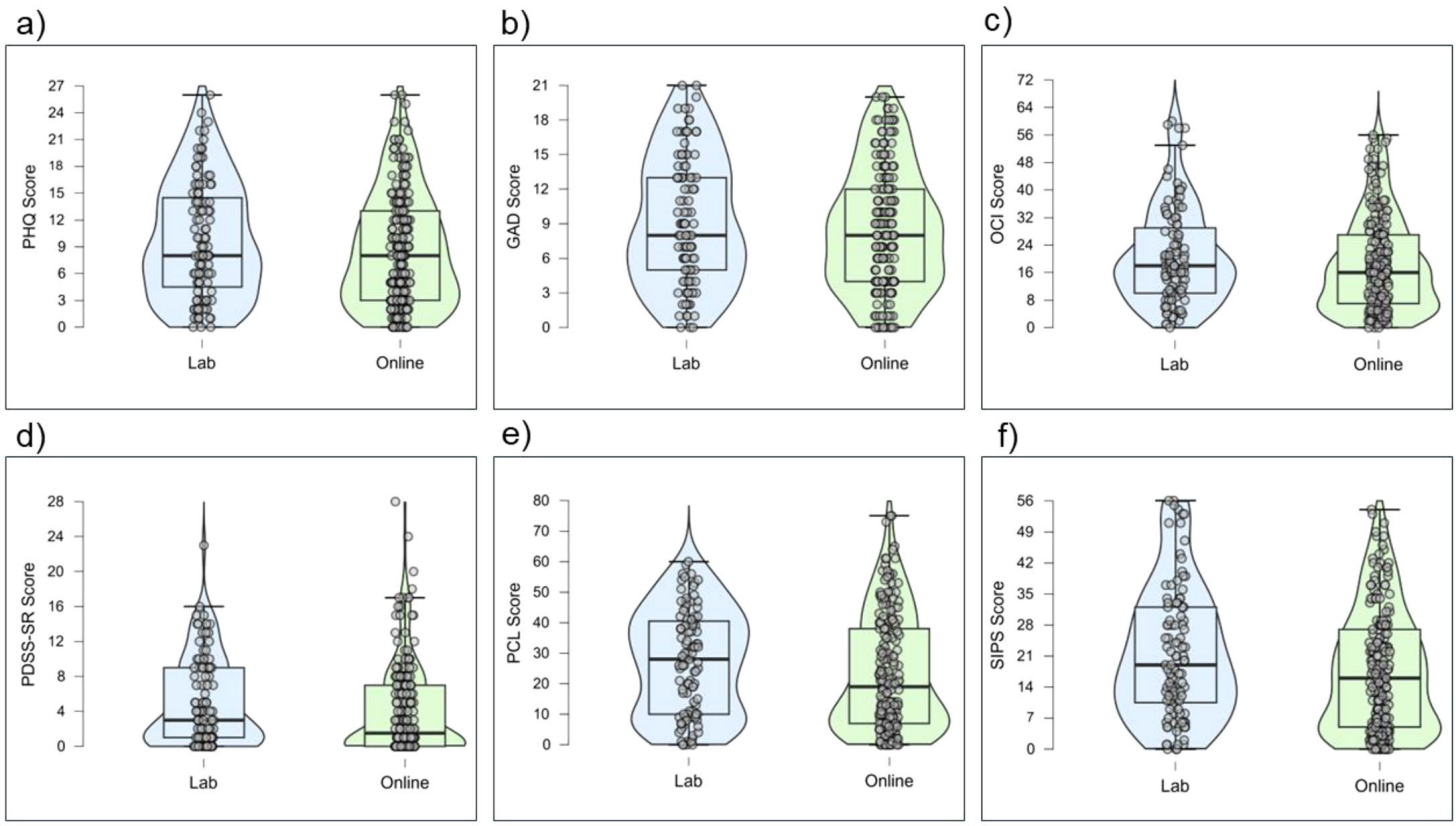
Violin plots of symptom measures by group: PHQ **(a)**, GAD **(b)**, OCI **(c)**, PDSS-SR **(d)**, PCL **(e)**, and SIPS **(f)**.

**Figure 8:**
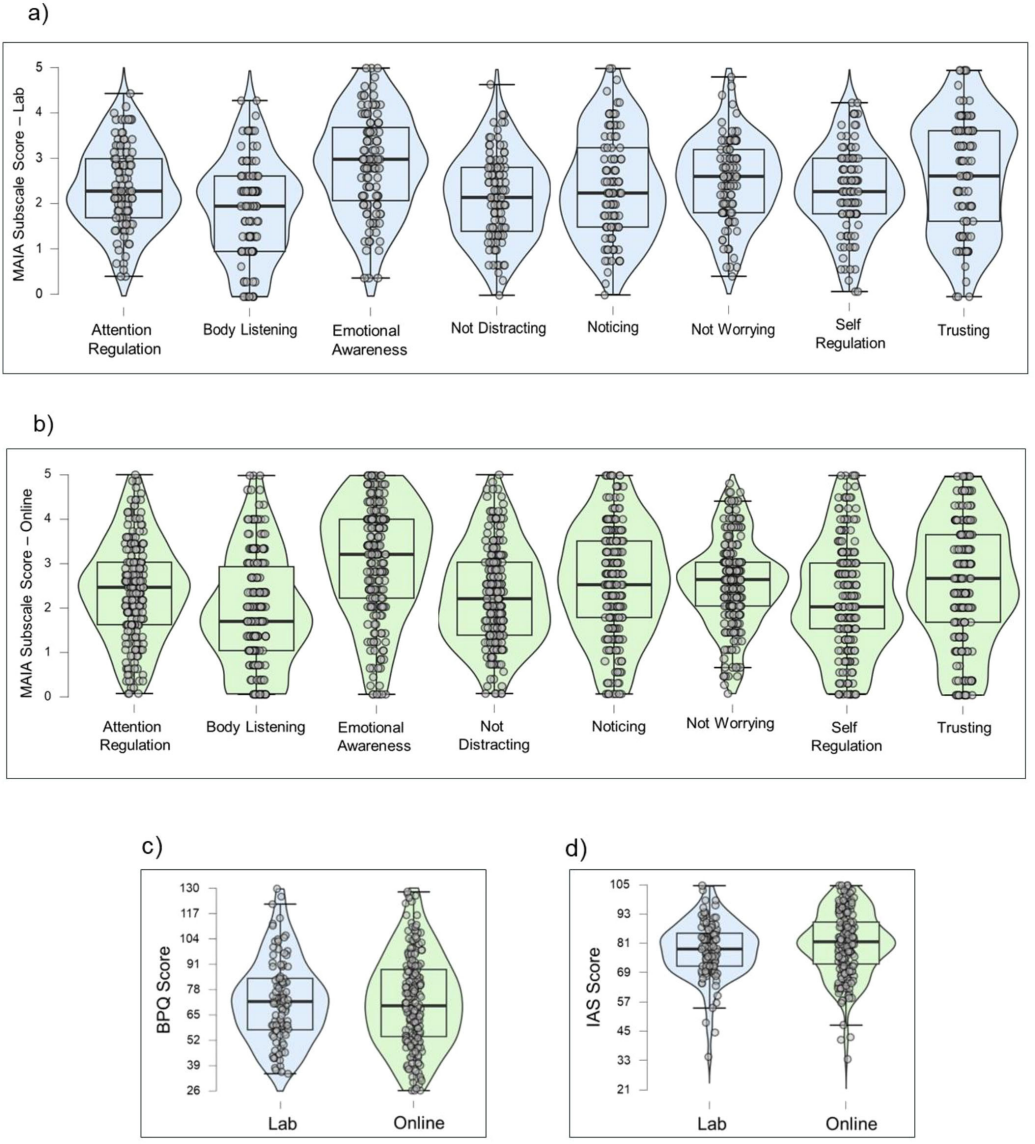
Violin plots of interoceptive measures by group: MAIA Subscales **(a, b)**, BPQ **(c)**, and IAS **(d)**.

The self-report measures display similar central tendencies and psychometric properties across the online and lab samples. Both groups consistently exhibited strong internal consistency. Overall, these descriptive statistics suggest the self-report instruments are reliable and perform as expected within this sample. **Figures 6**–**8** display violin plots of each anxiety trait measure, symptom measure and interceptive measure, split by group.

### Correlational analyses between anxiety-trait and interoception self–report measures

Parametric correlation coefficients were calculated among all scales. Correlations examined the associations between anxiety trait measures and interoception measures (see **Table 5**). As expected, there were significant positive relationships between the anxiety trait measures within this sample (*N* = 305). Scatterplots illustrating key associations are provided in **Supplementary Materials 3.1**, **3.2**.

**Table 5:** Correlation matrix illustrating the relationship between anxiety trait and self-report interoception questionnaires (N = 305).

Questionnaire/subscale	1	2	3	4	5	6	7	8	9	10	11	12	13
1.ASI	--												
2.IUS	.51**	--											
3.STAI-T	.56**	.68**	--										
4.BPQ	.32**	.10	.14*	--									
5.IAS	-.14*†	-.15**	-.15**	.08	--								
6.MAIA Noticing	.26**	.15*	.19**	.36**	.27**	--							
7.MAIA Not Distracting	-.28**	-.22**	-.27**	-.16**	.11*†	-.23**	--						
8.MAIA Not Worrying	-.60**	-.39**	-.44**	-.14*	.18**	-.09	.08	--					
9.MAIA Attention Reg.	-.10	-.10	-.07	.25**	.30**	.46**	-.09	.12*	--				
10.MAIA Emotional Awa.	.14*	.04	.09	.32**	.22**	.58**	-.19**	-.08	.55**	--			
11.MAIA Self-Regulation	-.19**	-.18**	-.24**	.09	.24**	.32**	.04	.15**	.65**	.57**	--		
12.MAIA Body Listening	.04	-.05	-.09	.17**	.25**	.45**	-.01	.01	.53**	.58**	.63**	--	
13.MAIA Trusting	-.16**	-.25**	-.26**	.06	.30**	.25**	.02	.13*	.57**	.51**	.60**	.54**	--

* and ** denote uncorrected significance at *p* <.05 and *p* <.01, respectively. † indicates the effect did not remain significant after Benjamini–Hochberg FDR correction (*q* = .05). ASI, Anxiety Sensitivity Index; BPQ, Body Perception Questionnaire; IAS, Interoceptive Accuracy Scale; IUS, Intolerance of Uncertainty Scale; MAIA, Multidimensional Assessment of Interoceptive Awareness; STAI-T, State Trait Anxiety Inventory – Trait. Correlational Analyses Between Symptom and Interoception Self – Report Measures.

Trait anxiety (STAI-T) showed significant negative correlations with MAIA subscales of ‘Not Distracting’ (*r* = -.27, *p* <.001), ‘Not Worrying’ (*r* = -.44, *p* <.001), ‘Self-Regulation’ (*r* = -.24, *p* <.001), and ‘Trusting’ (*r* = -.26, *p* <.001). A positive correlation was found between STAI-T and MAIA ‘Noticing’ (*r* = .19, p <.001). STAI-T scores were also weakly correlated with BPQ scores *(r* = .14, *p* <.014).

Anxiety sensitivity (ASI) exhibited significant negative correlations with MAIA subscales of ‘Not Distracting’ (*r* = -.28, *p* <.001), ‘Not Worrying’ (*r* = -.60, *p* <.001), and ‘Self-Regulation’ (*r* = -.19, *p* <.001), and ‘Emotional Awareness’ (*r* = .14, *p* <.015). A moderate positive association was also found between ASI and BPQ scores (*r* = .32, *p* <.001). A weaker negative association was found between ASI and IAS (*r* = -.14). However, this effect did not remain significant after correction for multiple comparisons.

Intolerance of uncertainty (IUS) was significantly negatively correlated with several MAIA subscales, including ‘Not Distracting’ (*r* = -.22, *p* <.001), ‘Not Worrying’ (*r* = -.39, *p* <.001), and ‘Self-Regulation’ (*r* = -.18, *p* = .002). A small negative relationship was identified between IU and self-reported interoceptive accuracy (IAS) (*r* = -.15, *p* = .009), and no relationship with BPQ.

### Correlational analyses between symptom and interoception self–report measures

**Table 6** outlines the associations between clinical symptom measures and self-report interoceptive measures. As expected, there were significant positive relationships between all symptom measures in this sample (*N* = 305) (*r* = .53 -.77, *p’s* <.001). Higher levels of GAD, PTSD and depression were significantly negatively correlated with the MAIA subscales ‘Not Distracting’ (see **Supplementary 3.3**), ‘Not Worrying’, ‘Self-Regulation’ and ‘Trusting’. Similarly, elevated higher panic and OCD symptoms were also significantly negatively correlated with the MAIA subscales of ‘Not Distracting’, ‘Not Worrying’, and ‘Trusting’. Social anxiety symptoms were primarily associated with lower scores on MAIA ‘Not Worrying’ (*r* -.33; *p* <.001).

**Table 6:** Correlation matrix illustrating the relationship between symptom questionnaires and self-report interoception questionnaire. (N = 305).

Questionnaire/Subscales	1	2	3	4	5	6	7	8	9	10	11	12	13	14	15	16
1. GAD	--															
2. OCI	.61**	--														
3. PCL	.74**	.64**	--													
4. PDSS	.60**	.53**	.66**	--												
5. PHQ	.72**	.56**	.77**	.54**	--											
6. SIPS	.53**	.55**	.57**	.51**	.49**	--										
7. BPQ	.16**	.23**	.20**	.24**	.16**	.28**	--									
8. IAS	-.15**	-.21**	-.25**	-.16**	-.22**	-.21**	.08	--								
9. MAIA Noticing	.21**	.21**	.20**	.26**	.14*	.19**	.36**	.27**	--							
10. MAIA Not Distracting	-.25**	-.28**	-.36**	-.25**	-.28**	-.27**	-.16**	.11*	-.23**	--						
11. MAIA Not Worrying	-.38**	-.33**	-.34**	-.28**	-.21**	-.33**	-.14*	.18**	-.09	.08	--					
12. MAIA Attention Reg.	-.09	.05	-.08	-.05	-.13*	-.04	.25**	.30**	.46**	-.09	.12*	--				
13. MAIA Emotional Awa.	.10	.12*†	.10	.15**	-.07	.07	.32**	.22**	.58**	-.19**	-.08	.55**	--			
14. MAIA Self-Regulation	-.20**	-.07	-.17**	-.11	-.27**	-.14*	.09	.24**	.32**	.04	.15**	.65**	.57**	--		
15. MAIA Body Listening	-.06	.05	-.02	.10	-.14*	.03	.17**	.25**	.45**	-.01	.01	.53**	.58**	.63**	--	
16. MAIA Trusting	-.26**	-.15**	-.22**	-.15*	-.29**	-.23**	.06	.30**	.25**	.02	.13*	.57**	.51**	.60**	.54**	--

* and ** denote uncorrected significance at *p* <.05 and *p* <.01, respectively. † indicates the effect did not remain significant after Benjamini–Hochberg FDR correction (*q* = .05). BPQ, Body Perception Questionnaire; GAD, Generalised Anxiety Disorder; IAS, Interoceptive Accuracy Scale; MAIA, Multidimensional Assessment of Interoceptive Awareness; OCI, Obsessive-Compulsive Inventory; PDDS-SR, Panic Disorder Severity Scale – Self Report; PCL, Post-Traumatic Stress Disorder Checklist; SIPS, Social Interaction Phobia Scale.

Higher interoceptive attention (BPQ) was positively associated with greater anxiety-related symptoms, particularly social anxiety (*r* = .28, *p* <.001), panic (*r* = .24, *p* <.001), OCD (*r* = .23, *p* <.001), and generalised anxiety (*r* = .16, *p = .*005). BPQ scores were also positively correlated with symptom severity of other emotional disorders including PTSD (*r* = .20, *p* <.001) and depression (*r* = .16, *p* = .005). Interoceptive accuracy (IAS) was significantly negatively correlated with all symptom measures, although the strength of this relationship was weak (*r* <.22, *p* ‘s = .001 -.008).

### Heartbeat perception task performance descriptive statistics

**Table 7** presents the descriptive statistics for the performance on the heartbeat perception tasks with the lab sample (*n* = 103). Data was normally distributed for both tasks.

**Table 7:** Descriptive statistics of heartbeat perception tasks.

Heartbeat Task	*N*	*M*	*SD*	Range	Skewness	Kurtosis
HBC Accuracy	103	0.72	0.17	0.23 – 0.98	-0.65	-0.28
HBC Confidence	103	43.48	19.80	0.17 – 89.83	-0.11	-0.42
HBC Insight	103	0.27	0.49	-0.80 – 0.97	-0.54	-0.75
HBD Accuracy	103	0.49	0.14	0.10 – 0.85	-0.23	0.23
HBD Confidence	103	53.63	16.95	8.65 – 95.05	-0.19	0.00
HBD Insight	103	0.51	0.14	0.11 – 0.87	-0.11	0.49

HBC, Heartbeat Counting; HBD, Heartbeat Detection.

**Figure 9:**
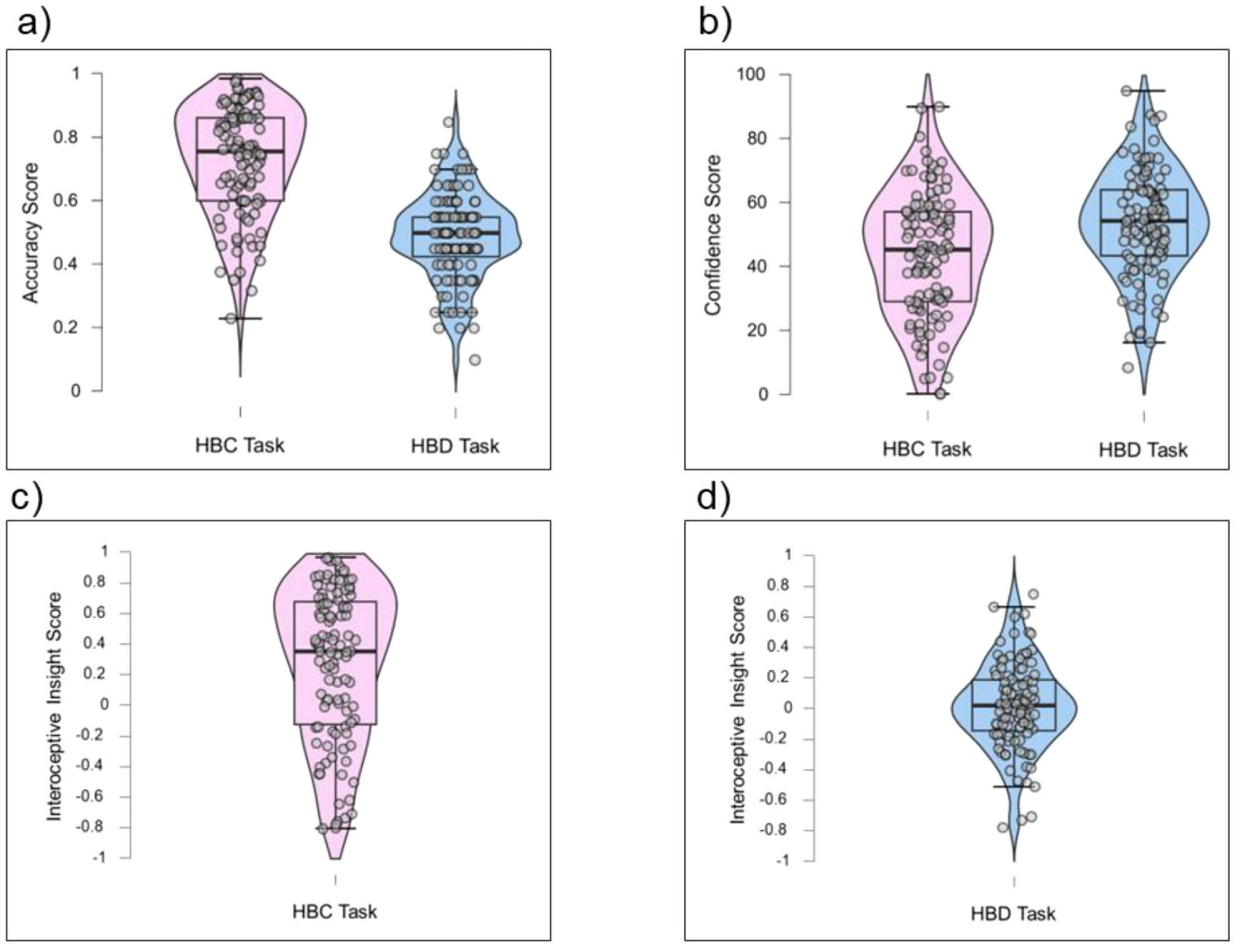
Violin plots of heartbeat perception task performance: Accuracy Score **(a)**, Confidence Score **(b)**, Interoceptive Insight Score **(c, d)**. N = 103.

Overall, participants demonstrated overall higher accuracy in the HCT task compared to the HDT task, suggesting heartbeat counting was easier than heartbeat detection. However, overall confidence scores were lower for the heartbeat counting task than the heartbeat detection task. **Figure 9** displays violin plots of interoceptive accuracy, confidence and insight metrics for each heartbeat perception task.

### Correlational analysis between anxiety trait and heartbeat perception task performance

Correlation analyses were conducted to explore associations between anxiety trait vulnerability factors (i.e., trait anxiety, AS and IU) and heartbeat perception task performance (see **Table 8**). For the heartbeat counting task, trait anxiety (STAI-T) was negatively associated with Heartbeat Counting (HBC) Confidence (*r* = -.26). Greater intolerance of uncertainty (IUS) was associated with increased HBC Insight (*r* = .20). However, both results did not withstand correction for multiple comparisons. No significant associations were found between trait measures and Heartbeat Detection (HBD) performance.

**Table 8:** Correlation matrix illustrating the relationship between trait questionnaires and heartbeat perception task performance. (N = 103).

Questionnaire/task	1	2	3	4	5	6	7	8	9
1. ASI	--								
2. IUS	.59**	--							
3. STAI-T	.46**	.65**	--						
4. HBC Accuracy	-.02	-.08	-.05	--					
5. HBC Confidence	-.07	-.08	-.26*†	.27**	--				
6. HBC Insight	-.02	.20*†	.17	.13	-.02	--			
7. HBD Accuracy	.12	-.01	-.01	-.02	-.08	.10	--		
8. HBD Confidence	-.18	-.13	-.11	.09	.54**	-.11	-.07	--	
9. HBD Insight	.03	-.05	.12	.03	-.14	.13	.02	-.06	--

* and ** denote uncorrected significance at *p* <.05 and *p* <.01, respectively. † indicates the effect did not remain significant after Benjamini–Hochberg FDR correction (*q* = .05). ASI, Anxiety Sensitivity Index; HBC, Heartbeat Counting; HBD, Heartbeat Detection; IUS, Intolerance of Uncertainty Scale; STAI-T, State-Trait Anxiety Inventory – Trait.

As expected, there were significant positive relationships between the anxiety-trait measures within the lab sample (*N* = 103). Higher intolerance of uncertainty (IUS) was strongly associated with greater trait anxiety (STAI-T) (*r* = .65, *p* <.001) and anxiety sensitivity (ASI) (*r* = .59, *p* <.001), consistent with theoretical models that conceptualise intolerance of uncertainty as a transdiagnostic cognitive vulnerability factor for anxiety disorders. Furthermore, trait anxiety (STAI-T) and anxiety sensitivity (ASI) were moderately correlated (*r* = .46, *p* <.001).

### Correlational analysis between symptom measures and heartbeat perception task performance

Correlation analyses were conducted to explore the relationships between symptom measures and performance on heartbeat perception tasks (see **Table 9**). No significant relationships emerged between symptom measures and heartbeat perception task metrics (i.e., interoceptive accuracy, confidence, and insight). As expected, strong positive correlations were observed between symptom questionnaires (*r* = .50 -.83, *p’s* <.001).

**Table 9:** Correlation matrix illustrating the relationship between symptom questionnaires and heartbeat perception task performance. (N = 103).

Questionnaire/Task	1	2	3	4	5	6	7	8	9	10	11	12
1. GAD	--											
2. OCI	.64**	--										
3. PCL	.83**	.56**	--									
4. PDSS-SR	.68**	.54**	.74**	--								
5. PHQ	.73**	.54**	.75**	.58**	--							
6. SIPS	.56**	.50**	.58**	.56**	.58**	--						
7. HBC Accuracy	.09	-.03	.08	.11	.02	-.08	--					
8. HBC Confidence	-.12	-.05	-.10	-.02	-.16	-.02	.27**	--				
9. HBC Insight	-.01	.02	-.02	-.16	-.02	-.12	.13	-.02	--			
10. HBD Accuracy	.01	-.07	.06	-.01	-.04	.08	-.02	-.08	.10	--		
11. HBD Confidence	-.09	-.05	-.01	-.02	-.11	-.04	.09	.54**	-.11	-.07	--	
12. HBD Insight	.01	-.05	.03	.09	.01	-.11	.03	-.14	.13	.02	-.06	--

* and ** denote uncorrected significance at *p* <no><.05</no> and *p* <.01, respectively. GAD, Generalised Anxiety Disorder; HBC, Heartbeat Counting; HBD, Heartbeat Detection; OCI, Obsessive-Compulsive Inventory; PDDS-SR, Panic Disorder Severity Scale – Self Report; PCL, Post-Traumatic Stress Disorder Checklist; PHQ, Patient health questionnaire; SIPS, social interaction phobia scale.Discussion.

The present study investigates the relationship between multiple facets of interoception, anxious-related traits, and anxiety-related symptoms. Given the heterogeneity of findings in the existing literature, hypotheses regarding these relationships were primarily exploratory, though some directional predictions were made based on prior research.

### H1. Anxiety-related traits and self-reported interoception

As predicted, anxiety-related traits were significantly associated with self-report measures of interoception. Specifically, trait anxiety was negatively related with MAIA subscales ‘Not Worrying’, ‘Not Distracting’, ‘Self- Regulation’ and ‘Trusting’ in line with previous research (e.g., 28, 32–34, 36). The strongest relationship was with ‘Not Worrying’ and ‘Trusting’, also consistent with literature (32, 34). This suggests individuals with higher trait anxiety may have difficulty not worrying about discomforting sensations, ignoring and distracting oneself from sensations of discomfit, regulating distress by attending to bodily sensations and trusting their body as a ‘safe place’. In contrast to previous findings, MAIA ‘Noticing’ was positively correlated with trait anxiety, although this relationship is weak. This novel finding may suggest individuals with higher trait anxiety tend to be more attuned to the presence of bodily sensations, but without the accompanying capacity to interpret or regulate them in adaptive ways in a non-clinical sample. These findings align with previous research that suggests higher trait anxiety is associated with greater self-reported sensitivity to internal bodily sensations (27, 37).

BPQ findings were consistent with those from the MAIA ‘Noticing’ subscale, also showing a positive correlation with trait anxiety. This may reflect the conceptual overlap between the two measures, as both assess subjective awareness of bodily sensations. However, the BPQ has been criticised as a proxy measure for anxiety symptoms, as it primarily captures awareness of aversive, anxiety-related bodily states. In contrast, the MAIA was specifically developed to assess more adaptive, regulatory aspects of interoception, such as mindfulness and coping behaviours (74). This distinction may help explain why some MAIA subscales show negative associations with anxiety, while the BPQ consistently shows positive relationships. These differential relationships highlight a need to distinguish between maladaptive and adaptive forms of interoception (90).

Similarly, AS was positively correlated with the BPQ and MAIA ‘Noticing’. Consistent with previous research, it showed the strongest negative correlation with MAIA ‘Not-Worrying’ (34, 38). This suggests that individuals with higher anxiety sensitivity tend to be more attuned to bodily sensations but are also more likely to interpret these sensations in a distressing way. As expected, individuals with lower AS may be less likely to worry when experiencing anxiety-related bodily sensations, such as increased heartrate, shortness of breath or chest constrictions.

Given the scarcity of research examining the relationship between IU and interoception, the present study adopted an exploratory approach. Findings revealed that IU was negatively correlated with the MAIA ‘Not Distracting’ and ‘Trusting’ subscales, with the strongest negative correlation observed for ‘Not Worrying’, as reported in existing research (49). These results suggest individuals with higher IU may be less likely to trust their bodily sensations, more prone to worry about them, and more inclined to distract themselves from these signals rather than engage with them adaptively. However, no significant relationship was found between IU and interoceptive attention, as measured by the BPQ, while only a weak positive association emerged with the MAIA Noticing subscale, which also assesses awareness of bodily sensation. This suggests IU may not be related to the extent to which individuals attend to bodily sensations, but rather how individuals may interpret and respond to those sensations. This is further supported by the observed negative association between IU and self-reported interoceptive accuracy, as measured by the IAS. Together, these findings highlight the potential role of higher IU in altered interoceptive processing, characterised by negative beliefs about bodily signals (i.e., interpreting them as threatening), and reduced perceived accuracy in detecting these signals.

### H2. Anxiety-related traits and heartbeat perception tasks

Results from the heartbeat perception tasks found no relationship between cardiac interoceptive accuracy and any of the anxiety-related traits examined. This adds to the body of evidence that suggests interoceptive accuracy does not play a significant role in trait anxiety (28, 30, 31, 85), anxiety sensitivity (42) and provides novel findings for IU. This supports the theoretical model that anxious traits are more closely linked to interceptive beliefs (i.e., how much they believe they notice bodily sensations), rather than behavioural measures of interoceptive accuracy (8, 91).

Notably, IU was at trend positively correlated with interoceptive insight on the HBC task, but not on the HBD task. This discrepancy may be due to inherent differences in task requirements (92). Whilst both tasks are presumed to assume perception of internal sensations, the detection task is a force-choice discrimination task which requires the integration of internal and external stimuli. As such, HBD tasks are often presumed to be more difficult than the HBC task (8, 93).

### H3. Anxiety-related symptoms and self-reported interoception

Significant relationships were found between all symptom measures and the IAS and BPQ. BPQ scores were positively correlated with symptom severity, whereas IAS scores were negatively correlated. This suggests that individuals who report poorer interoceptive accuracy tend to experience higher levels of anxiety-related symptoms, with the strongest association observed for PTSD symptoms. Conversely, individuals with greater interoceptive attention tend to report higher symptom severity. These findings suggest that individuals may be highly attuned to their bodily sensations but lack confidence in the accuracy of their perceptions. This aligns with models suggesting that heightened bodily awareness may contribute to increased symptom severity through hypervigilance and misinterpretation of bodily sensations (21, 43).

Supporting this, negative relationships between all symptom measures and the MAIA ‘Trusting’ subscale suggest that individuals with more severe symptoms may struggle to trust their bodily sensations, particularly those with panic symptoms, where this association was strongest. This pattern is consistent with theories of panic disorder, which emphasise the catastrophic misinterpretation of bodily signals (94–96). Several MAIA subscales were significantly negative associated with symptom measures. For example, PTSD symptoms were most strongly associated with lower scores on the ‘Not Distracting’ subscale. This is in line with the well-established role of avoidance in PTSD, particularly the avoidance of internal cues that may trigger trauma memories (55). This highlights that individuals with PTSD may actively disengage from bodily sensations in an effort to prevent distressing flashbacks or anxiety, reflecting interoceptive avoidance.

### H4. Anxiety-related symptoms and heartbeat perception tasks

No significant correlations were found between symptom measures and metrics derived from the HBC and HBD tasks (i.e., interoceptive accuracy, confidence, and interoceptive insight). These findings are unexpected, and challenge theoretical models that position interoception as central to anxiety disorder symptoms (e.g., 21, 43). This adds to the growing body of research reporting differential associations between interoceptive processes and anxiety symptoms, suggesting that psychopathology may not be characterised by global interoceptive differences (97). These mixed findings may be due to differences in how interoception is conceptualised and methodological variations, underscoring the need for more standardised approaches to studying interoception in psychopathology (2, 98). Moreover, the absence of significant associations may be partly attributable to the non-clinical sample and the reliance on self-reported symptoms. As illustrated in the violin plots (see **Figure 7**), most symptom scores, apart from GAD, were clustered toward the lower end of the scale, which may have limited the variability needed to detect associations with behavioural interoception.

## Discussion

This study applies a multidimensional model of interoception to examine both transdiagnostic anxious traits and symptom dimensions across anxiety-related disorders. Strengths include the combined use of self-report and heartbeat perception tasks, a relatively large sample size, and inclusion of a range of anxiety constructs. By aligning interoceptive measures with a contemporary multidimensional framework, the study helps clarify which interoceptive domains (e.g., accuracy, attention, beliefs) are most relevant to anxiety.

Limitations should also be acknowledged. The present study focuses exclusively on cardiac interoception, therefore results may not generalise to other domains such as respiratory or gastric interoception, which have also shown associations with anxiety when measured (e.g., 12). 39 found performance on inspiratory resistance tasks does not necessarily transfer to cardiac tasks, highlighting that interoceptive abilities may be modality specific. Future research should adopt multimodal approaches to determine whether observed associations are unique to cardiac interoception or reflect broader interoceptive mechanisms.

Secondly, despite widespread use in interoceptive research, the heartbeat perception tasks have been criticised for their validity (99, 100). The HBD task can produce mean accuracies around chance level (50%), as observed in the present study and reported in other studies using comparable (101) or greater numbers of trials (102). This suggests that the task may be particularly challenging for participants. Therefore, the individual difference results here should be interpreted with caution. Consequently, further methodological refinement and technological development are needed to improve the sensitivity, reliability, and validity of heartbeat perception tasks in interoceptive research. Furthermore, it has been argued that HBC performance can be influenced by non-interoceptive factors such as time estimation, knowledge of heart rate and response bias (84, 100, 103). Prior knowledge of heart rate may be particularly relevant given the growing prevalence of heart rate-monitoring wearables, which could potentially confound performance on the HBC task (104). Desmedt et al. (105) demonstrated that modifying task instructions to ask participants not to guess their heartbeats reduced average HCT performance by 50%, suggesting HCT performance may rely on cognitive strategies rather than true interoceptive accuracy. To address these concerns and minimise estimation bias and chance-level responding, future studies should assess participants’ baseline knowledge of their heart rate and time estimation abilities, as implemented in some studies (103, 106, 107). This would help clarify the extent to which top-down influences affect task performance and disentangle interoceptive ability from other cognitive factors.

Furthermore, the present study did not observe a significant relationship between HBC and HBD performance (see **Supplementary Material**), consistent with previous research (108, 109). Meta-analytic evidence suggests performance on the HBC and HBD tasks is only weakly associated, indicating such tasks may capture distinct constructs (92). This raises questions about whether these tasks index a shared interoceptive ability or instead tap into distinct facets of interoception.

Lastly, the sample that was recruited in the current study was intended to reflect a general non-clinical population. However, participants were not excluded based on psychiatric conditions, including personality disorders. In the sample that was recruited, 40-45% met cut-offs for clinically relevant symptoms such as moderate anxiety and depression (see **Supplementary Table 2**). While there was high reporting of clinically relevant symptoms, it is important to note that anxiety and depression symptoms are relatively common, particularly amongst young people (110). Furthermore, the questionnaires used are for screening purposes only. A diagnostic clinical interview conducted by a trained clinician would be a more appropriate tool to identify diagnosis status. On this basis, in this data set we could not address the impact of mental health status on the result.

In light of the rapidly rising global prevalence of anxiety disorders (58), continued research into interoception as a transdiagnostic factor is warranted. Future studies should extend beyond single measurement paradigms and adopt a systematic, multidimensional assessment of interoception, including exploration of understudied bodily domains beyond the cardiac axis. Such an approach will help to delineate the mechanistic role of interoception in anxiety-related disorders, enhance the development of targeted clinical interventions, and enhance understanding of the real-world relevance and translational potential of interoceptive processes (111).

## Conclusion

The present study explored the relationship between interoception and anxiety-related traits and symptoms using a multidimensional framework. By combining self-report and behavioural measures, it aimed to capture distinct facets of interoception to address the gaps in the literature. While some findings aligned with theoretical predictions, highlighting the role of interoceptive beliefs and attention in anxiety, other findings, particularly those involving behavioural task performance, were less consistent. The lack of relationship between heartbeat perception tasks and self-reported experiences highlights ongoing concerns about measurement validity (88, 111). Overall, this research provides novel findings related to anxious-traits and interoception and supports the move towards multidimensional approaches to interoception. Findings highlight the importance of considering both subjective and behavioural measures of interoceptive processes when investigating its role in anxiety-related processes.

Future research should prioritise methodological standardisation, for example by using consistent operationalisations of interoceptive dimensions (accuracy, attention, beliefs, and insight) and using validated behavioural and self-report measures across studies to improve comparability. Including clinically severe and diverse populations, such as those recruited from secondary and tertiary services, would also strengthen generalisability. Finally, clarifying the translational relevance of interoceptive differences could involve examining whether these processes predict treatment response, act as mechanisms of change, or represent novel intervention targets. Such advances may guide the tailoring of treatments to individual interoceptive profiles and enhance therapeutic efficacy.

## Data Availability

The raw data supporting the conclusions of this article will be made available by the authors, without undue reservation.

## References

[1] Chen WG Schloesser D Arensdorf AM Simmons JM Cui C Valentino R . The emerging science of interoception: Sensing, integrating, interpreting, and regulating signals within the self. Trends Neurosci. (2021) 44:3–16. doi: 10.1016/j.tins.2020.10.007. PMID: 33378655 PMC7780231

[2] Khalsa SS Adolphs R Cameron OG Critchley HD Davenport PW Feinstein JS . Interoception and mental health: A roadmap. Biol Psychiatry: Cogn Neurosci Neuroimaging. (2018) 3:501–13. doi: 10.1016/j.bpsc.2017.12.004. PMID: 29884281 PMC6054486

[3] Craig AD . How do you feel? Interoception: The sense of the physiological condition of the body. Nat Rev Neurosci. (2002) 3:655–66. doi: 10.1038/nrn894. PMID: 12154366

[4] Critchley HD Garfinkel SN . Interoception and emotion. Curr Opin Psychol. (2017) 17:7–14. doi: 10.1016/j.copsyc.2017.04.020. PMID: 28950976

[5] Quigley KS Kanoski S Grill WM Barrett LF Tsakiris M . Functions of interoception: From energy regulation to experience of the self. Trends Neurosci. (2021) 44:29–38. doi: 10.1016/j.tins.2020.09.008. PMID: 33378654 PMC7780233

[6] Tsakiris M Critchley H . Interoception beyond homeostasis: affect, cognition and mental health. Phil Trans R Soc. (2016) 371:20160002. doi: 10.1098/rstb.2016.0002. PMID: 28080961 PMC5062092

[7] Brewer R Murphy J Bird G . Atypical interoception as a common risk factor for psychopathology: A review. Neurosci Biobehav Rev. (2021) 130:470–508. doi: 10.1016/j.neubiorev.2021.07.036. PMID: 34358578 PMC8522807

[8] Suksasilp C Garfinkel SN . Towards a comprehensive assessment of interoception in a multi-dimensional framework. Biol Psychol. (2022) 168:108262. doi: 10.1016/j.biopsycho.2022.108262. PMID: 35026353

[9] Garfinkel SN Seth AK Barrett AB Suzuki K Critchley HD . Knowing your own heart: Distinguishing interoceptive accuracy from interoceptive awareness. Biol Psychol. (2015) 104:65–74. doi: 10.1016/j.biopsycho.2014.11.004. PMID: 25451381

[10] Schandry R . Heart beat perception and emotional experience. Psychophysiology. (1981) 18:483–8. doi: 10.1111/j.1469-8986.1981.tb02486.x. PMID: 7267933

[11] Whitehead WE Drescher VM Heiman P Blackwell B . Relation of heart rate control to heartbeat perception. Biofeedback Self-regulation. (1977) 2:371–92. doi: 10.1007/bf00998623. PMID: 612350

[12] Harrison OK Köchli L Marino S Luechinger R Hennel F Brand K . Interoception of breathing and its relationship with anxiety. Neuron. (2021) 109:4080–93. doi: 10.1016/j.neuron.2021.09.045. PMID: 34672986 PMC8691949

[13] Porges SW . Body Perception Questionnaire. Laboratory of Developmental Assessment, University of Maryland (1993).

[14] Khalsa SS Lapidus RC . Can interoception improve the pragmatic search for biomarkers in psychiatry? Front Psychiatry. (2016) 7:121. doi: 10.3389/fpsyt.2016.00121. PMID: 27504098 PMC4958623

[15] Pang J Tang X Li H Hu Q Cui H Zhang L . Altered interoceptive processing in generalized anxiety disorder—A heartbeat-evoked potential research. Front Psychiatry. (2019) 10:616. doi: 10.3389/fpsyt.2019.00616. PMID: 31543837 PMC6739601

[16] Verdonk C Teed AR White EJ Ren X Stewart JL Paulus MP . Heartbeat-evoked neural response abnormalities in generalized anxiety disorder during peripheral adrenergic stimulation. Neuropsychopharmacology. (2024) 49:1246–54. doi: 10.1038/s41386-024-01806-5. PMID: 38291167 PMC11224228

[17] Boswell JF Farchione TJ Sauer-Zavala S Murray HW Fortune MR Barlow DH . Anxiety sensitivity and interoceptive exposure: A transdiagnostic construct and change strategy. Behav Ther. (2013) 44:417–31. doi: 10.1016/j.beth.2013.03.006. PMID: 23768669 PMC3727659

[18] Carleton RN . Fear of the unknown: One fear to rule them all? J Anxiety Disord. (2016) 41:5–21. doi: 10.1016/j.janxdis.2016.03.011. PMID: 27067453

[19] McEvoy PM Mahoney AE . To be sure, to be sure: Intolerance of uncertainty mediates symptoms of various anxiety disorders and depression. Behav Ther. (2012) 43:533–45. doi: 10.1016/j.beth.2011.02.007. PMID: 22697442

[20] Paulus DJ Talkovsky AM Heggeness LF Norton PJ . Beyond negative affectivity: A hierarchical model of global and transdiagnostic vulnerabilities for emotional disorders. Cogn Behav Ther. (2015) 44:389–405. doi: 10.1080/16506073.2015.1017529. PMID: 25734894

[21] Domschke K Stevens S Pfleiderer B Gerlach AL . Interoceptive sensitivity in anxiety and anxiety disorders: An overview and integration of neurobiological findings. Clin Psychol Rev. (2010) 30:1–11. doi: 10.1016/j.cpr.2009.08.008. PMID: 19751958

[22] Pollatos O Herbert BM Kaufmann C Auer DP Schandry R . Interoceptive awareness, anxiety and cardiovascular reactivity to isometric exercise. Int J Psychophysiol. (2007) 65:167–73. doi: 10.1016/j.ijpsycho.2007.03.005. PMID: 17449123

[23] Pollatos O Traut-Mattausch E Schroeder H Schandry R . Interoceptive awareness mediates the relationship between anxiety and the intensity of unpleasant feelings. J Anxiety Disord. (2007) 21:931–43. doi: 10.1016/j.janxdis.2006.12.004. PMID: 17257810

[24] De Pascalis V Alberti ML Pandolfo R . Anxiety, perception, and control of heart rate. Perceptual Motor Skills. (1984) 59:203–11. doi: 10.2466/pms.1984.59.1.203. PMID: 6493936

[25] Kutscheidt K Dresler T Hudak J Barth B Blume F Ethofer T . Interoceptive awareness in patients with attention-deficit/hyperactivity disorder (ADHD). ADHD Attention Deficit Hyperactivity Disord. (2019) 11:395–401. doi: 10.1007/s12402-019-00299-3. PMID: 30937850

[26] Duschek S Werner NS del Paso GAR Schandry R . The contributions of interoceptive awareness to cognitive and affective facets of body experience. J Individ Dif. (2015) 36(2):110–118. doi: 10.1027/1614-0001/a000165, PMID: 37214235

[27] Garfinkel SN Tiley C O’Keeffe S Harrison NA Seth AK Critchley HD . Discrepancies between dimensions of interoception in autism: Implications for emotion and anxiety. Biol Psychol. (2016) 114:117–26. doi: 10.1016/j.biopsycho.2015.12.003. PMID: 26724504

[28] Slotta T Witthöft M Gerlach AL Pohl A . The interplay of interoceptive accuracy, facets of interoceptive sensibility, and trait anxiety: a network analysis. Pers Individ Dif. (2021) 183:111133. doi: 10.1016/j.paid.2021.111133. PMID: 41936479

[29] Werner NS Schweitzer N Meindl T Duschek S Kambeitz J Schandry R . Interoceptive awareness moderates neural activity during decision-making. Biol Psychol. (2013) 94:498–506. doi: 10.1016/j.biopsycho.2013.09.002. PMID: 24076035

[30] Adams KL Edwards A Peart C Ellett L Mendes I Bird G . The association between anxiety and cardiac interoceptive accuracy: A systematic review and meta-analysis. Neurosci Biobehav Rev. (2022) 140:104754. doi: 10.1016/j.neubiorev.2022.104754. PMID: 35798125

[31] Desmedt O Van Den Houte M Walentynowicz M Dekeyser S Luminet O Corneille O . A systematic review and meta-analysis on the association between heartbeat counting task performance and mental disorders and their risk factors among adults. (2020). doi: 10.31219/osf.io/h3by9, PMID: 41064916

[32] Bornemann B Herbert BM Mehling WE Singer T . Differential changes in self-reported aspects of interoceptive awareness through 3 months of contemplative training. Front Psychol. (2015) 5:1504. doi: 10.3389/fpsyg.2014.01504. PMID: 25610410 PMC4284997

[33] Ferentzi E Olaru G Geiger M Vig L Köteles F Wilhelm O . Examining the factor structure and validity of the multidimensional assessment of interoceptive awareness. J Pers Assess. (2021) 103:675–84. doi: 10.1080/00223891.2020.1813147. PMID: 32955947

[34] Mehling WE Price C Daubenmier JJ Acree M Bartmess E Stewart A . The multidimensional assessment of interoceptive awareness (MAIA). PLoS One. (2012) 7:e48230. doi: 10.1371/journal.pone.0048230. PMID: 23133619 PMC3486814

[35] Mehling W . Differentiating attention styles and regulatory aspects of self-reported interoceptive sensibility. Philos Trans R Soc B Biol Sci. (2016) 371:20160013. doi: 10.1098/rstb.2016.0013. PMID: 28080970 PMC5062101

[36] Borg C Chouchou F Dayot-Gorlero J Zimmerman P Maudoux D Laurent B . Pain and emotion as predictive factors of interoception in fibromyalgia. J Pain Res. (2018), 823–35. doi: 10.2147/JPR.S152012. PMID: 29719416 PMC5914549

[37] Palser ER Palmer CE Galvez-Pol A Hannah R Fotopoulou A Kilner JM . Alexithymia mediates the relationship between interoceptive sensibility and anxiety. PLoS One. (2018) 13:203212. doi: 10.1371/journal.pone.0203212. PMID: 30212484 PMC6136731

[38] Tünte MR Petzke TM Brand S Murphy J Witthöft M Hoehl S . He who seeks finds (bodily signals): German validation of the interoceptive attention scale (IATS) and its relationship with subclinical psychopathology. J Pers Assess. (2024) 106:787–97. doi: 10.1080/00223891.2024.2316236. PMID: 38478969 PMC7616536

[39] Harrison OK Köchli L Marino S Marlow L Finnegan SL Ainsworth B . Gender differences in the association between anxiety and interoceptive insight. Eur J Neurosci. (2025) 61:16672. doi: 10.22541/au.171647268.89829982/v1. PMID: 39804235 PMC11728262

[40] Reiss S Peterson RA Gursky DM McNally RJ . Anxiety sensitivity, anxiety frequency and the prediction of fearfulness. Behav Res And Ther. (1986) 24:1–8. doi: 10.1016/0005-7967(86)90143-9. PMID: 3947307

[41] Eley TC Gregory AM Clark DM Ehlers A . Feeling anxious: A twin study of panic/somatic ratings, anxiety sensitivity and heartbeat perception in children. J Child Psychol Psychiatry. (2007) 48:1184–91. doi: 10.1111/j.1469-7610.2007.01838.x. PMID: 18093023

[42] Körmendi J Ferentzi E Petzke T Gál V Köteles F . Do we need to accurately perceive our heartbeats? Cardioceptive accuracy and sensibility are independent from indicators of negative affectivity, body awareness, body image dissatisfaction, and alexithymia. PLoS One. (2023) 18:e0287898. doi: 10.1371/journal.pone.0287898. PMID: 37406011 PMC10321613

[43] Paulus MP Stein MB . Interoception in anxiety and depression. Brain Structure Funct. (2010) 214:451–63. doi: 10.1007/s00429-010-0258-9. PMID: 20490545 PMC2886901

[44] Stewart SH Buffett-Jerrott SE Kokaram R . Heartbeat awareness and heart rate reactivity in anxiety sensitivity: A further investigation. J Anxiety Disord. (2001) 15:535–53. doi: 10.1016/s0887-6185(01)00080-9. PMID: 11764311

[45] Sturges LV Goetsch VL . Psychophysiological reactivity and heartbeat awareness in anxiety sensitivity. J Anxiety Disord. (1996) 10:283–94. doi: 10.1016/0887-6185(96)00012-6

[46] Gualtieri I Parisi I Bortolini T Porciello G Panasiti MS . Exploring the relationship between anxiety sensitivity, interoceptive sensibility and psychopathology: A network analysis approach. Int J Cogn Behav Ther. (2025) 18:256–83. doi: 10.1007/s41811-025-00235-6. PMID: 41933263

[47] Melhi E Zarani F Panaghi L Harirchian MH Farsijani N Hosseini Nasr SZ . How interoceptive awareness and mindfulness relate to anxiety sensitivity: Mediating role of emotion dysregulation. Ment Health: Res Pract. (2023) 2:24–33. doi: 10.22034/MHRP.2024.429008.1024. PMID: 36190387

[48] Morriss J . Psychological mechanisms underpinning change in intolerance of uncertainty across anxiety-related disorders: New insights for translational research. Neurosci Biobehav Rev. (2025) 173:106138. doi: 10.1016/j.neubiorev.2025.106138, PMID: 40216169

[49] Bijsterbosch JM Hasenack B van Rooijen B Sternheim LC Boelen PA Dijkerman HC . Intolerable feelings of uncertainty within the body: Associations between interoceptive awareness, intolerance of uncertainty, and body dissatisfaction. J Adolescence. (2023) 95:1678–88. doi: 10.1002/jad.12237. PMID: 37655512

[50] Freeston M Komes J . Revisiting uncertainty as a felt sense of unsafety: The somatic error theory of intolerance of uncertainty. J Behav Ther Exp Psychiatry. (2023) 79:101827. doi: 10.1016/j.jbtep.2022.101827. PMID: 36512913

[51] Wilson LA Scarfo J Jones ME Rehm IC . The relationship between sensory phenomena and interoception across the obsessive–compulsive spectrum: a systematic review. BMC Psychiatry. (2025) 25:162. doi: 10.1186/s12888-024-06441-4. PMID: 39994601 PMC11849306

[52] Zoellner LA Craske MG . Interoceptive accuracy and panic. Behav Res And Ther. (1999) 37:1141–58. doi: 10.1016/s0005-7967(98)00202-2. PMID: 10596462

[53] Stevens S Gerlach AL Cludius B Silkens A Craske MG Hermann C . Heartbeat perception in social anxiety before and during speech anticipation. Behav Res And Ther. (2011) 49:138–43. doi: 10.1016/j.brat.2010.11.009. PMID: 21147477

[54] Reinhardt KM Zerubavel N Young AS Gallo M Ramakrishnan N Henry A . A multi-method assessment of interoception among sexual trauma survivors. Physiol Behav. (2020) 226:113108. doi: 10.1016/j.physbeh.2020.113108. PMID: 32721494

[55] Schmitz M Back SN Seitz KI Harbrecht NK Streckert L Schulz A . The impact of traumatic childhood experiences on interoception: disregarding one’s own body. Borderline Pers Disord And Emotion Dysregulation. (2023) 10:5. doi: 10.1186/s40479-023-00212-5. PMID: 36788573 PMC9930318

[56] Eggart M Lange A Binser MJ Queri S Müller-Oerlinghausen B . Major depressive disorder is associated with impaired interoceptive accuracy: A systematic review. Brain Sci. (2019) 9:131. doi: 10.3390/brainsci9060131. PMID: 31174264 PMC6627769

[57] Saltafossi M Heck D Kluger DS Varga S . Common threads: Altered interoceptive processes as transdiagnostic mechanisms across affective and anxiety disorders. J Affect Disord 369, 244–254. (2024). doi: 10.1016/j.jad.2024.09.135. PMID: 39321982

[58] Chen S Huang W Zhang M Song Y Zhao C Sun H . Dynamic changes and future trend predictions of the global burden of anxiety disorders: Analysis of 204 countries and regions from 1990 to 2021 and the impact of the COVID-19 pandemic. EClinicalMedicine. (2025) 79:103014. doi: 10.1016/j.eclinm.2024.103014. PMID: 39834715 PMC11743809

[59] Faul F Erdfelder E Buchner A Lang A-G . Statistical power analyses using G* Power 3.1: Tests for correlation and regression analyses. Behav Res Methods. (2009) 41:1149–60. doi: 10.3758/BRM.41.4.1149. PMID: 19897823

[60] Morriss J Wake S Elizabeth C Van Reekum CM . I doubt it is safe: A meta-analysis of self-reported intolerance of uncertainty and threat extinction training. Biol Psychiatry Global Open Sci. (2021) 1:171–9. doi: 10.1016/j.bpsgos.2021.05.011. PMID: 36325301 PMC9616306

[61] Pfeifer G Garfinkel SN van Praag CDG Sahota K Betka S Critchley HD . Feedback from the heart: Emotional learning and memory is controlled by cardiac cycle, interoceptive accuracy and personality. Biol Psychol. (2017) 126:19–29. doi: 10.1016/j.biopsycho.2017.04.001. PMID: 28385627

[62] Carleton RN Norton MPJ Asmundson GJ . Fearing the unknown: A short version of the Intolerance of Uncertainty Scale. J Anxiety Disord. (2007) 21:105–17. doi: 10.1016/j.janxdis.2006.03.014. PMID: 16647833

[63] Zsido AN Teleki SA Csokasi K Rozsa S Bandi SA . Development of the short version of the spielberger state—trait anxiety inventory. Psychiatry Res. (2020) 291:113223. doi: 10.1016/j.psychres.2020.113223. PMID: 32563747

[64] Spielberger CD . Manual for the State-Trait Anxiety Inventory (Self-Evaluation Questionnaire). Palo Alto: Consulting Psychologists Press (1970).

[65] Vujanovic AA Arrindell WA Bernstein A Norton PJ Zvolensky MJ . Sixteen-item anxiety sensitivity index: Confirmatory factor analytic evidence, internal consistency, and construct validity in a young adult sample from the Netherlands. Assessment. (2007) 14:129–43. doi: 10.1177/1073191106295053. PMID: 17504886

[66] Kroenke K Spitzer RL Williams JB . The PHQ‐9: validity of a brief depression severity measure. J Gen Internal Med. (2001) 16:606–13. doi: 10.1046/j.1525-1497.2001.016009606.x. PMID: 11556941 PMC1495268

[67] Kroenke K Spitzer RL Williams JB Löwe B . The patient health questionnaire somatic, anxiety, and depressive symptom scales: a systematic review. Gen Hosp Psychiatry. (2010) 32:345–59. doi: 10.1016/j.genhosppsych.2010.03.006. PMID: 20633738

[68] Spitzer RL Kroenke K Williams JB Löwe B . A brief measure for assessing generalized anxiety disorder: the GAD-7. Arch Internal Med. (2006) 166:1092–7. doi: 10.1001/archinte.166.10.1092. PMID: 16717171

[69] Foa EB Huppert JD Leiberg S Langner R Kichic R Hajcak G . The Obsessive-Compulsive Inventory: Development and validation of a short version. psychol Assess. (2002) 14:485. doi: 10.1037/1040-3590.14.4.485. PMID: 12501574

[70] Houck PR Spiegel DA Shear MK Rucci P . Reliability of the self‐report version of the panic disorder severity scale. Depression Anxiety. (2002) 15:183–5. doi: 10.1002/da.10049. PMID: 12112724

[71] Weathers FW Litz BT Keane TM Palmieri PA Marx BP Schnurr PP . The PTSD Checklist for DSM-5 (PCL-5). (2013). Scale available from the National Center for PTSD at: https://www.ptsd.va.gov.

[72] Blevins CA Weathers FW Davis MT Witte TK Domino JL . The posttraumatic stress disorder checklist for DSM‐5 (PCL‐5): Development and initial psychometric evaluation. J Traumatic Stress. (2015) 28:489–98. doi: 10.1002/jts.22059. PMID: 26606250

[73] Carleton RN Collimore KC Asmundson GJ McCabe RE Rowa K Antony MM . Refining and validating the social interaction anxiety scale and the social phobia scale. Depression Anxiety. (2009) 26:71–81. doi: 10.1002/da.20480. PMID: 19152346

[74] Mehling WE Acree M Stewart A Silas J Jones A . The multidimensional assessment of interoceptive awareness, version 2 (MAIA-2). PLoS One. (2018) 13:e0208034. doi: 10.1371/journal.pone.0208034. PMID: 30513087 PMC6279042

[75] Eggart M Todd J Valdés-Stauber J . Validation of the Multidimensional Assessment of Interoceptive Awareness (MAIA-2) questionnaire in hospitalized patients with major depressive disorder. PLoS One. (2021) 16:253913. doi: 10.1371/journal.pone.0253913. PMID: 34170963 PMC8232409

[76] Fekih-Romdhane F Malaeb D Fawaz M Chammas N Soufia M Obeid S . Psychometric properties of an arabic translation of the multidimensional assessment of interoceptive awareness (MAIA-2) questionnaire in a non-clinical sample of Arabic-speaking adults. BMC Psychiatry. (2023) 23:577. doi: 10.1186/s12888-023-05067-2. PMID: 37558996 PMC10410825

[77] Fiskum C Eik-Nes TT Abdollahpour Ranjbar H Andersen J Habibi Asgarabad M . Interoceptive awareness in a Norwegian population: Psychometric properties of the Multidimensional Assessment of Interoceptive Awareness (MAIA) 2. BMC Psychiatry. (2023) 23:489. doi: 10.1186/s12888-023-04946-y. PMID: 37430262 PMC10331976

[78] Scheffers M Coenen J Moeijes J de Haan A van Busschbach J Bellemans T . The Multidimensional Assessment of Interoceptive Awareness, version 2 (MAIA-2): psychometric properties in a Dutch non-clinical sample. BMC Psychol. (2024) 12:53. doi: 10.1186/s40359-024-01553-8. PMID: 38287385 PMC10826081

[79] Cabrera A Kolacz J Pailhez G Bulbena‐Cabre A Bulbena A Porges SW . Assessing body awareness and autonomic reactivity: Factor structure and psychometric properties of the Body Perception Questionnaire‐Short Form (BPQ‐SF). Int J Methods Psychiatr Res. (2018) 27:1596. doi: 10.1002/mpr.1596. PMID: 29193423 PMC6877116

[80] Betka S Pfeifer G Garfinkel S Prins H Bond R Sequeira H . How do self‐assessment of alexithymia and sensitivity to bodily sensations relate to alcohol consumption? Alcoholism: Clin Exp Res. (2018) 42:81–8. doi: 10.1111/acer.13542. PMID: 29094768

[81] Murphy J Brewer R Plans D Khalsa SS Catmur C Bird G . Testing the independence of self-reported interoceptive accuracy and attention. Q J Exp Psychol. (2020) 73:115–33. doi: 10.1177/1747021819879826. PMID: 31519137

[82] Payne R Symeonides C Webb D Maxwell S . Pulse transit time measured from the ECG: an unreliable marker of beat-to-beat blood pressure. J Appl Physiol. (2006) 100:136–41. doi: 10.1152/japplphysiol.00657.2005. PMID: 16141378

[83] Wiens S Palmer SN . Quadratic trend analysis and heartbeat detection. Biol Psychol. (2001) 58:159–75. doi: 10.1016/s0301-0511(01)00110-7. PMID: 11600243

[84] Ring C Brener J . Heartbeat counting is unrelated to heartbeat detection: A comparison of methods to quantify interoception. Psychophysiology. (2018) 55:13084. doi: 10.1111/psyp.13084. PMID: 29633292

[85] Desmedt O Van Den Houte M Walentynowicz M Dekeyser S Luminet O Corneille O . How does heartbeat counting task performance relate to theoretically-relevant mental health outcomes? A meta-analysis. Collabra: Psychol. (2022) 8:33271. doi: 10.1525/collabra.33271. PMID: 41538050

[86] Green DM Swets JA . Signal Detection Theory and Psychophysics Vol. 1. . New York: Wiley (1966).

[87] Qualtrics . Qualtrics XM Platform (Version April 2024). Qualtrics (2024). Available online at: https://www.qualtrics.com.

[88] Murphy J Brewer R Coll M-P Plans D Hall M Shiu SS . I feel it in my finger: Measurement device affects cardiac interoceptive accuracy. Biol Psychol. (2019) 148:107765. doi: 10.1016/j.biopsycho.2019.107765, PMID: 31518599

[89] IBM Corp . IBM SPSS Statistics for Windows (Version 29.0.2.0) [Computer Software] (2023). Available online at: https://www.ibm.com/products/spss-statistics.

[90] Trevisan DA Mehling WE McPartland JC . Adaptive and maladaptive bodily awareness: Distinguishing interoceptive sensibility and interoceptive attention from anxiety‐induced somatization in autism and alexithymia. Autism Res. (2021) 14:240–7. doi: 10.1002/aur.2458. PMID: 33336935

[91] Murphy J Catmur C Bird G . Classifying individual differences in interoception: Implications for the measurement of interoceptive awareness. Psychonomic Bull Rev. (2019) 26:1467–71. doi: 10.3758/s13423-019-01632-7. PMID: 31270764 PMC6797703

[92] Hickman L Seyedsalehi A Cook JL Bird G Murphy J . The relationship between heartbeat counting and heartbeat discrimination: A meta-analysis. Biol Psychol. (2020) 156:107949. doi: 10.1016/j.biopsycho.2020.107949. PMID: 32911018

[93] Murphy J Brewer R Catmur C Bird G . Interoception and psychopathology: A developmental neuroscience perspective. Dev Cognit Neurosci. (2017) 23:45–56. doi: 10.1016/j.dcn.2016.12.006. PMID: 28081519 PMC6987654

[94] Clark DM . A cognitive approach to panic. Behav Res Ther. (1986) 24:461–70. doi: 10.1016/0005-7967(86)90011-2. PMID: 3741311

[95] Clark DM Salkovskis PM Öst L-G Breitholtz E Koehler KA Westling BE . Misinterpretation of body sensations in panic disorder. J Consulting Clin Psychol. (1997) 65:203. doi: 10.1037//0022-006x.65.2.203. PMID: 9086683

[96] Ehlers A Breuer P . Increased cardiac awareness in panic disorder. J Abnormal Psychol. (1992) 101:371. doi: 10.1037//0021-843x.101.3.371. PMID: 1500594

[97] Schoeller FA Zhang B Garcia T Reggente N . There is no such thing as interoception. Front Psychol. (2025) 16:1488415. doi: 10.3389/fpsyg.2025.1488415. PMID: 39995425 PMC11847802

[98] Desmedt O Luminet O Maurage P Corneille O . Discrepancies in the definition and measurement of human interoception: A comprehensive discussion and suggested ways forward. Perspect psychol Sci. (2025) 20:76–98. doi: 10.1177/17456916231191537. PMID: 37642084

[99] Corneille O Desmedt O Zamariola G Luminet O Maurage P . A heartfelt response to Zimprich et al.(2020), and Ainley et al.(2020)’s commentaries: Acknowledging issues with the HCT would benefit interoception research. Biol Psychol. (2020) 152:107869. doi: 10.1016/j.biopsycho.2020.107869. PMID: 32061686

[100] Zamariola G Maurage P Luminet O Corneille O . Interoceptive accuracy scores from the heartbeat counting task are problematic: Evidence from simple bivariate correlations. Biol Psychol. (2018) 137:12–7. doi: 10.1016/j.biopsycho.2018.06.006. PMID: 29944964

[101] Minenko I Limonova A Sukmanova A Kutsenko V Nazarova M Ershova A . Comparison of three behavioral cardioception tasks and heartbeat evoked potentials in the same group of healthy volunteers. Sci Rep. (2025) 15:35773. doi: 10.1038/s41598-025-08779-5. PMID: 41087368 PMC12521517

[102] Kleckner IR Wormwood JB Simmons WK Barrett LF Quigley KS . Methodological recommendations for a heartbeat detection‐based measure of interoceptive sensitivity. Psychophysiology. (2015) 52:1432–40. doi: 10.1111/psyp.12503. PMID: 26265009 PMC4821012

[103] Murphy J Millgate E Geary H Ichijo E Coll M-P Brewer R . Knowledge of resting heart rate mediates the relationship between intelligence and the heartbeat counting task. Biol Psychol. (2018) 133:1–3. doi: 10.1016/j.biopsycho.2018.01.012. PMID: 29378285

[104] Prieto-Avalos G Cruz-Ramos NA Alor-Hernandez G Sánchez-Cervantes JL Rodriguez-Mazahua L Guarneros-Nolasco LR . Wearable devices for physical monitoring of heart: a review. Biosensors. (2022) 12:292. doi: 10.3390/bios12050292. PMID: 35624593 PMC9138373

[105] Desmedt O Luminet O Corneille O . The heartbeat counting task largely involves non-interoceptive processes: Evidence from both the original and an adapted counting task. Biol Psychol. (2018) 138:185–8. doi: 10.1016/j.biopsycho.2018.09.004. PMID: 30218689

[106] Haruki Y Kaneko K Ogawa K . No gender difference in cardiac interoceptive accuracy: Potential psychophysiological contributors in heartbeat counting task. BMC Psychol. (2025) 13:176. doi: 10.1186/s40359-025-02432-6. PMID: 40022207 PMC11871792

[107] Sakuragi M Umeda S . How the heart shapes the mind: The role of cardiac interoception in the interaction between bodily reactions and self-related thoughts. Acta Psychol (Amst). (2025) 261:105931. doi: 10.1016/j.actpsy.2025.105931, PMID: 41265322

[108] Forkmann T Scherer A Meessen J Michal M Schächinger H Vögele C . Making sense of what you sense: Disentangling interoceptive awareness, sensibility and accuracy. Int J Psychophysiol. (2016) 109:71–80. doi: 10.1016/j.ijpsycho.2016.09.019. PMID: 27702644

[109] Schulz A Lass-Hennemann J Sütterlin S Schächinger H Vögele C . Cold pressor stress induces opposite effects on cardioceptive accuracy dependent on assessment paradigm. Biol Psychol. (2013) 93:167–74. doi: 10.1016/j.biopsycho.2013.01.007. PMID: 23354518

[110] Barker MM Beresford B Bland M Fraser LK . Prevalence and incidence of anxiety and depression among children, adolescents, and young adults with life-limiting conditions: A systematic review and meta-analysis. JAMA Pediatr. (2019) 173:835–44. doi: 10.1001/jamapediatrics.2019.1712. PMID: 31282938 PMC6618774

[111] Heim N Bobou M Tanzer M Jenkinson PM Steinert C Fotopoulou A . Psychological interventions for interoception in mental health disorders: A systematic review of randomized‐controlled trials. Psychiatry Clin Neurosci. (2023) 77:530–40. doi: 10.1111/pcn.13576. PMID: 37421414 PMC7615164

