# Supplementary material for: Exploring the role of interoception in anxious traits and symptoms
Source: Front Psychiatry. 2026 Apr 29;17:1769315. doi: 10.3389/fpsyt.2026.1769315 (PMC13167933; doi:10.3389/fpsyt.2026.1769315)
Supplement: Supplementary file 1 [file SupplementaryFile1.docx]

**Exploring The Role of Interoception in Anxious Traits and Symptoms**

**Supplementary Materials**

1. **Verbatim Task Instructions**

“For this study, you will also be asked to complete some questionnaires, two computer-based tasks, and heartrate/blood pressure readings. The entire session will take around one hour in total.”

‘’I’d like you to complete a series of questionnaires on the computer using the keyboard. These questionnaires will ask you about your demographics, and your feelings and moods. Please read through the instructions for each questionnaire carefully, as sometimes they are asking you about how you feel in general or how you feel over a specified amount of time (like a week/month). Go with your gut instinct and don’t think too much about your answers. It’s completely confidential, I can’t see your answers so please answer honestly. I will be in the room across the corridor, please let me know when you are done by simply saying ‘I’m done’”.

| □ 1. Demographic Information (age, sex, ethnicity)  □ 2. Intolerance of Uncertainty Scale (IUS-12; Carleton et al., 2007)  □ 3. Trait Anxiety Shortened (STAI-T; Zsido et al, 2020)  □ 4. Generalised Anxiety Disorder Questionnaire (GAD-7; Spitzer et al., 2006)  □ 5. Patient Health Questionnaire (PHQ-9; Kroenke et al, 2001)  □ 6. Obsessive Compulsive Inventory - Revised (OCI-R; Foa et al., 2002)  □ 7. Panic Disorder Severity Scale (PDSS-SR; Shear et al., 1997)  □ 8. Social Interaction Phobia Scale (SIPS; Menatti et al., 2015)  □ 9. Posttraumatic Stress Disorder Checklist for DSM-5 (PCL-5; Weathers et al., 2013)  □ 10. Multidimensional Assessment of Interoceptive Awareness-v2) (MAIA-v2; Mehling et al., 2018)  □ 11. Body Awareness Subscale of the Body Perception Questionnaire-Short Form (BPQ-SF; Cabrera et al., 2018)  □ 12. Interoceptive Accuracy Scale (IAS, Murphy et al., 2020)  □ 13. Anxiety Sensitivity Index (Reiss et al., 1986) |
| --- |

“For the cardiac perception tasks, you will be wearing headphones and a pulse oximeter which will record your pulse continuously.

Please wipe the fingers of your non-dominant hand and put the pulse oximeter on your index or middle finger (the fingers might change throughout the tasks in case of signal loss).

With your dominant hand, you will need to provide confidence ratings during the task using pen and paper.

Sit close to the table, resting your elbow on the table. It is very important that you keep your arm and hand very still during the trials to avoid signal loss. Signal loss is common and may well happen on a few occasions throughout the task. We try to reduce the occurrence by asking you to keep your hand still”

While I get the tasks set up, please sit back in your chair and relax a bit. …

We will now begin with the heartbeat counting task and run a couple of practice trials.

*Heartbeat Counting Task*

- Through the headphones you will hear a voice saying “Start”. After this, please silently count your heartbeat without manually checking your pulse.
- At the end of the trial, the voice will say “Stop”, and you will be asked to report how many heartbeats you counted.
- We then ask you to rate your confidence on the piece of paper by placing a X on the horizontal line after each trial.
- There are 6 short trials for this task.

*Heartbeat Discrimination (Detection) Task*

- Through the headphones, you will hear your own heartbeat presented as auditory tones.
- The tones are played either in sync with your own heartbeat, or slightly out of sync.
- Please pay attention to the auditory tones and evaluate whether they are played on or off your own heartbeat.
- At the end of each trial, you will be required to decide whether the tones were presented in or out of sync with your heartbeat.
- We then ask you to rate your confidence on the piece of paper by placing a X on the horizontal line after each trial.
- There are 20 trials in this task, each trial lasting 20 seconds.
- As before, we begin with a couple of practice trials.

1. **Correlational Analysis Between Self-Report Interoceptive Measures and Heartbeat Perception Task Performance**

Parametric correlation analyses were conducted to explore associations between self-report interoceptive measures and heartbeat perception task performance (see Table 1).

Regarding the heartbeat counting task, HBC Confidence positively correlated with subjective interoceptive accuracy (*IAS)* (*r* = .20, *p* = .043), and multiple MAIA subscales, including ‘Noticing’ (*r* = .22, *p* = .025), ‘Not Worrying’ (*r* = .19, *p* = .049), ‘Body Listening’ (*r* = .21, *p* = .032). Stronger associations were observed between HBC Confidence and ‘Attention Regulation’ (*r* = .28, *p* = .005) (see supplement 3.4), ‘Self-Regulation’ (*r* = .24, *p* = .016), and ‘Trusting’ (*r* = .27, *p* = .006). HBC awareness was negatively correlated with MAIA ‘Body Listening’ (*r* = -.26, *p* = .009). Regarding the HBD task, HBD Confidence was positively associated with the IAS (*r* = .21, *p* = .027) and MAIA ‘Noticing’ (*r* = .21, *p* = .027), ‘Not Worrying’ (*r* = .26, *p* = .007) and ‘Emotional Awareness’ (*r* = .27, *p* = .007) subscales (see supplement 3.5).

Significant correlations emerged between the self-reported interoception measures. Interoceptive attention (BPQ) was positively correlated with interoceptive accuracy (IAS) (*r* = .29, *p* < .001), and interoceptive beliefs (MAIA) ‘Noticing’ (*r* = .25, *p* < .001), ‘Attention Regulation’ (*r* = .33, *p* < .001), ‘Emotional Awareness’ (*r* = .27, *p* < .001), and ‘Body Listening’ (*r* = .25, *p* < .001). IAS was also positively associated with multiple aspects of interoceptive beliefs, including MAIA ‘Noticing’ (*r* = .31, *p* < .001), ‘Attention Regulation’ *(r* = .24, *p* < .001), ‘Emotional Awareness’ (*r* = .21, *p* < .001), ‘Self-Regulation’ *(r* = .27, *p* < .001), and ‘Body Listening’ (*r* = .26, *p* < .001).

**Table 1** Correlation Matrix Illustrating the Relationship Between Interoception Questionnaires and Heartbeat Perception Task Performance (N = 103)

| Questionnaire / Task | 1 | 2 | 3 | 4 | 5 | 6 | 7 | 8 | 9 | 10 | 11 | 12 | 13 | 14 | 15 | 16 |
| --- | --- | --- | --- | --- | --- | --- | --- | --- | --- | --- | --- | --- | --- | --- | --- | --- |
| 1. BPQ | -- |  |  |  |  |  |  |  |  |  |  |  |  |  |  |  |
| 2. IAS | .29** | -- |  |  |  |  |  |  |  |  |  |  |  |  |  |  |
| 3. MAIA Noticing | .25* | .31** | -- |  |  |  |  |  |  |  |  |  |  |  |  |  |
| 4. MAIA Not Distracting | -.18 | .08 | -.37** | -- |  |  |  |  |  |  |  |  |  |  |  |  |
| 5. MAIA Not Worrying | -.08 | .09 | -.12 | .18 | -- |  |  |  |  |  |  |  |  |  |  |  |
| 6. MAIA Attention Reg. | .33** | .24* | .34** | -.28** | .19 | -- |  |  |  |  |  |  |  |  |  |  |
| 7. MAIA Emotional Awa. | .27** | .21* | .53** | -.24* | .01 | .42** | -- |  |  |  |  |  |  |  |  |  |
| 8. MAIA Self-Regulation | .15 | .27** | .18 | -.05 | .20* | .58** | .44** | -- |  |  |  |  |  |  |  |  |
| 9. MAIA Body Listening | .25* | .26** | .29** | .07 | .08 | .45** | .47** | .59** | -- |  |  |  |  |  |  |  |
| 10. MAIA Trusting | .18 | .20* | 013 | -.02 | .19 | .55** | .35** | .54** | .53** | -- |  |  |  |  |  |  |
| 11. HBC Accuracy | .10 | .11 | .03 | -.01 | .12 | .10 | .10 | .19 | -.02 | -.01 | -- |  |  |  |  |  |
| 12. HBC Confidence | .03 | .20* | .22* | .01 | .19* | .28** | .19 | .24* | .21* | .27** | .27** | -- |  |  |  |  |
| 13. HBC Insight | -.14 | -.01 | -.02 | -.13 | -.01 | -.05 | .01 | -.02 | -.26** | -.15 | .13 | -.02 | -- |  |  |  |
| 14. HBD Accuracy | -.07 | -.07 | -.06 | -.09 | -.00 | -.06 | -.05 | .05 | -.03 | .01 | -.02 | -.08 | .10 | -- |  |  |
| 15. HBD Confidence | -.02 | .22* | .22* | .10 | .26** | .17 | .27** | .18 | .19 | .13 | .09 | .54** | -.11 | -.07 | -- |  |
| 16. HBD Insight | .08 | -.07 | .07 | -.10 | -.09 | .02 | -.03 | -.11 | .07 | -.08 | .03 | -.14 | .13 | .02 | -.06 | -- |

*Note.* * = *p* < .05, ** = *p* < .01. Two-tailed. BPQ = Body Perception Questionnaire; IAS = Interoceptive Accuracy Scale; MAIA = Multidimensional Assessment of Interoceptive Awareness; HBC = Heartbeat Counting; HBD = Heartbeat Detection.

**Correlational Analysis Between Heartbeat Perception Task Performance Variables**

Correlation analyses between heartbeat perception task performance variables revealed a significant positive correlation between *HBC Accuracy* and *HBC Confidence (r* = .27, *p* = .007). As expected, HBD Confidence was strongly associated with HBC Confidence (*r* = .54, p < .001). However, HBC and HBD interoceptive accuracy scores were not correlated between tasks, or HBC and HBD insight scores (see Table 1).

1. **Scatterplots of Key Relationships**

**3.1 Scatterplot of Anxiety Sensitivity & Self-reported Interoceptive** **Attention**


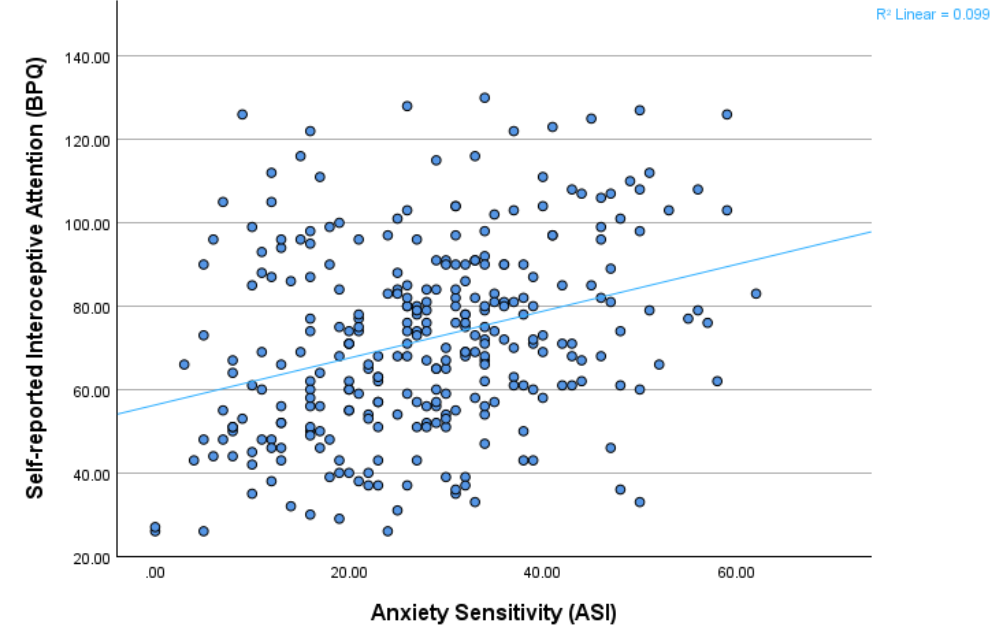


**3.2 Scatterplot of Intolerance of Uncertainty & MAIA ‘Not Worrying’**


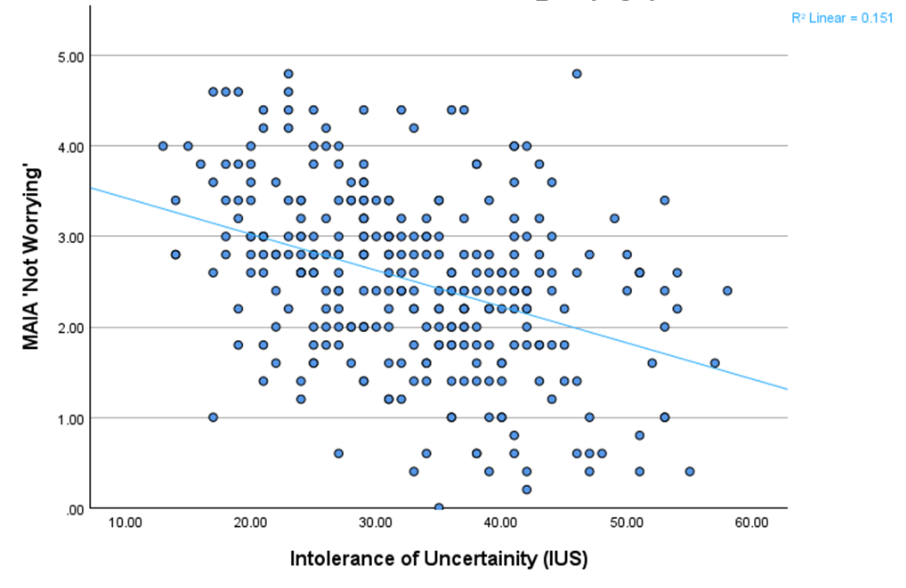


**3.3 Scatterplot of PTSD Symptom Severity & MAIA ‘Not Distracting’**


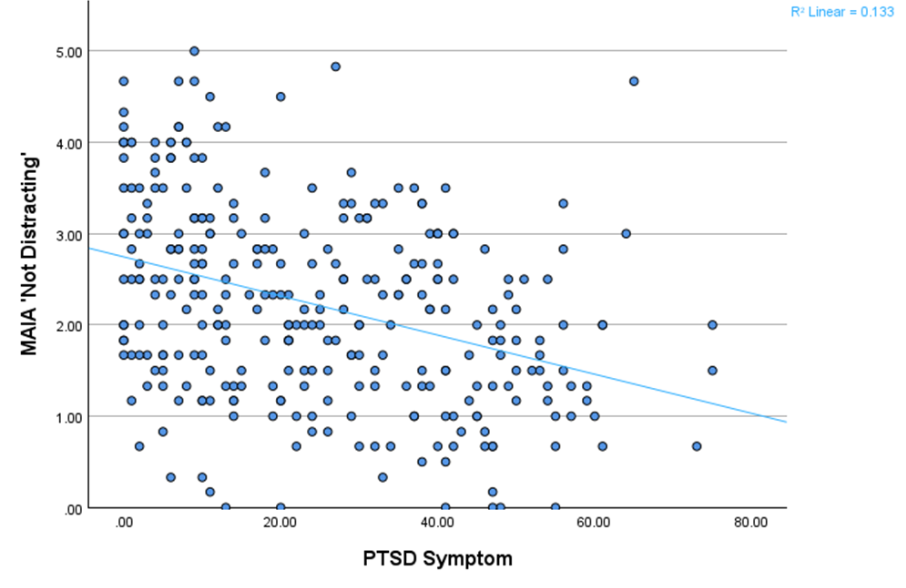


**3.4 Scatterplot of Heartbeat Counting Confidence & MAIA ‘Attention Regulation’**


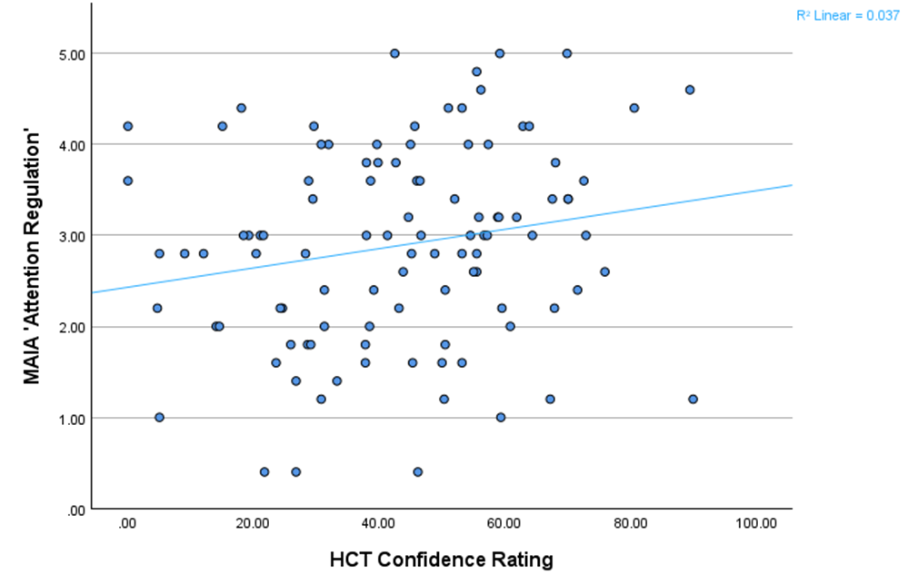


- 1. **Scatterplot of Heartbeat Detection Confidence & MAIA ‘Emotional Awareness’**


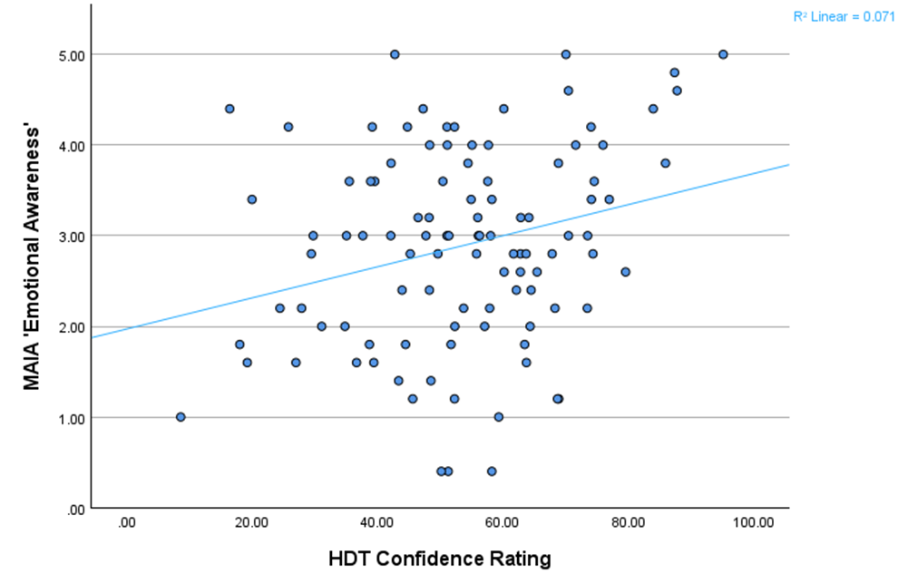


| **Table 2**  Proportion of participants exceeding clinical cut-offs across measures | | |
| --- | --- | --- |
| Measure | n (%) | Cut-off |
| PHQ-9 | 127 (41.64%) | ≥10 |
| GAD-7 | 134 (41.33%) | ≥10 |
| OCR-R | 136 (45.30%) | ≥21 |
| PDSS-SR | 75 (25.00%) | ≥8 |
| PCL-5 | 120 (40.00%) | ≥31 |
| SIPS | 137 (45.67%) | ≥24 |
| Note. PHQ-9 = Patient Health Questionnaire-9; GAD-7 = Generalized Anxiety Disorder-7; OCR-R = Obsessive-Compulsive Revised; PDSS-SR = Panic Disorder Severity Scale–Self Report; PCL-5 = PTSD Checklist for DSM-5; SIPS = Social Interaction Phobia Scale. | | |
